# Enhancing Relative Binding Free Energy Calculation with Grand Canonical Monte Carlo, Water-swap Monte Carlo, Terminal-flip Monte Carlo and Replica Exchange Solute Tempering

**DOI:** 10.1021/acs.jctc.6c00593

**Published:** 2026-06-15

**Authors:** Chenggong Hui, Bert L. de Groot

**Affiliations:** Computational Biomolecular Dynamics Group, 28282 Max Planck Institute for Multidisciplinary Sciences, Am Fassberg 11, 37077 Göttingen, Germany

## Abstract

Alchemical free energy calculations, particularly free energy perturbation (FEP), have become routine in drug design. When molecular dynamics (MD) is employed as the sampling method in FEP, accurately sampling water molecules deeply buried in protein binding pockets remains challenging, despite their critical role in protein–ligand interactions. We implemented three enhanced sampling methods, Grand Canonical Monte Carlo (GCMC), water-swap Monte Carlo (water-swap MC), and replica exchange with solute tempering (REST2). GCMC or water-swap MC accelerates the hydration state equilibration in buried pockets and REST2 accelerates the sampling of ligand conformations. Combining REST2 with GCMC or water-swap MC enables accurate calculation of ligand relative binding free energies. In the benchmark on the water set, our package achieved an RMSE of 0.92 kcal/mol, compared to 0.86 kcal/mol from FEP+ and 1.60 kcal/mol from OpenFE. This development provides the scientific community with an open-source sampling engine for robust and accurate FEP calculations.

## Graphical abstract

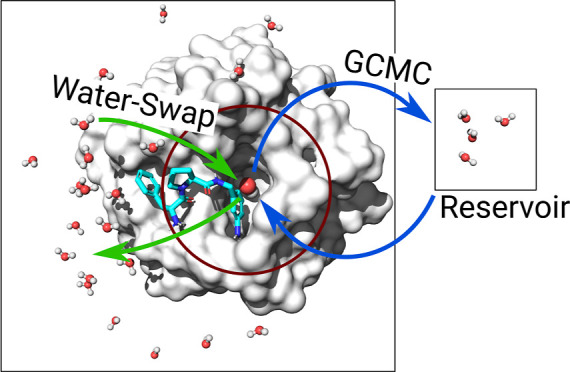

## 1 Introduction

In structure-based lead optimization, designing a ligand that can occupy the water-binding sites is a useful strategy. If a new ligand can occupy a water-binding site and replace bound-water-mediated interactions with the protein, the resulting entropy gain can improve binding affinity by up to 2 kcal/mol.^1^ Bound water molecules are widely found at the protein–ligand interface,^2^ but some of them are challenging to sample in classical molecular dynamics (MD). This kind of interior water can have a residence time of hundreds of microseconds,^3^ which is much longer than the typical simulation time of all-atom MD. Thus, accounting for the presence or displacement can be crucial for accurate affinity predictions.

Two major classes of methods have been developed to improve sampling of buried hydration sites beyond conventional MD without prior knowledge of the number of water in the binding site. The first class is grand canonical Monte Carlo (GCMC), where simulations are performed in a grand canonical (μVT) ensemble that keeps the chemical potential fixed while allowing number of water (particles) to fluctuation through insertion/deletion Monte Carlo moves.^4–6^ The second class is water-swap Monte Carlo (water-swap MC) in fixed-particle-number (typically *NPT* ensemble) simulations.^6–11^ Representative implementations now span both commercial and open-source ecosystems: Schrödinger’s GPU-accelerated GCMC-FEP + workflow,^4,12^ the OpenMM grand module for GCMC/MD simulations,^5^ and BLUES-style NCMC water hopping/translation protocols.^7,13^ Recent comparative studies further show that while these water-move methods can substantially improve hydration-state sampling, efficiency and equilibrated occupancy can remain method- and protocol-dependent.^6^ Despite this progress, both open-source tools grand and BLUES are still primarily single-Hamiltonian water-sampling engines and do not natively provide an integrated multi-λ alchemical production workflow for alchemical relative binding free energy (RBFE). If there is prior knowledge about the number of water molecules in the binding sites, the binding site water can also be coupled with λ coordinates and efficiently sampled.^14^ For example, the binding site water can be sampled with accelerated enveloping distribution sampling simultaneously with alchemical modifications of a ligand.^15^

In this work, we report the implementation of two water sampling methods—GCMC and water-swap MC, together with ligand sampling methods REST2 and terminal-flip Monte Carlo. The package was implemented in OpenMM,^16^ fully leveraging GPU acceleration. We begin by discussing the underlying theory behind GCMC, water-swap MC, and REST2. This is followed by benchmark results on the water set from Ross et al., comprising eight protein systems and 76 protein–ligand binding free energies (Δ*G*).^4^ We further demonstrate that key water-binding sites can be efficiently sampled with both GCMC and water-swap MC. We also found that in the thrombin system, terminal-flip Monte Carlo can efficiently sample the dihedrals and raise accuracy. Finally, we compare two force–field combinations: Amber14SB/TIP3P and Amber19SB/OPC.

## 2 Methods

### 2.1 Grand Canonical Monte Carlo (GCMC)

#### 2.1.1 Theory

In the grand canonical ensemble, volume **V**, temperature **T**, and chemical potential μ (for each exchangeable species, e.g., water) are fixed. Thus, the grand canonical ensemble is referred to as the μVT ensemble.^17^ The chemical potential μ is the free-energy change associated with adding one particle to the system. The probability of being a specific microstate with configuration **x** and particle number **N** is given by the probability distribution

1 $$p\left(x,N\right)=\frac{\exp\left(-\beta \left(H\left(x\right)-\mu N\right)\right)}{Zh^{3N}N!}$$

In eq 1, *H* denotes the Hamiltonian of the system, *h* is the Planck constant, β = *1*/*k*_B_ *T* is the inverse temperature and *Z* is the grand canonical partition function, which is

2 $$Z=\sum_{N=0}^{\infty }\frac{\int \exp\left(-\beta \left(H\left(x\right)-\mu N\right)\right)dx}{h^{3N}N!}$$

This partition function (eq 2) can be seen as the sum of a set of canonical ensembles with different *N*. If we want to sample between different *N*, we can apply the Metropolis–Hastings algorithm. For systems with 1 type of particle, after integrating out the momenta and simplifying the configurational weight, we have

3 $$\pi \left(N,x\right)\propto \frac{1}{N!}{\left(\frac{V}{\Lambda^{3}}\right)}^{3}\exp\left(-\beta U\left(x\right)+\beta \mu N\right)$$

In eq 3, Λ is the thermodynamic wavelength of the particle. For classical particle systems, we can further simplify the configurational weight to

4 $$\pi \left(N,x\right)\propto \frac{1}{N!}{\left(\frac{V}{V^{{}^{\circ}}}\right)}^{3}\exp\left(-\beta U\left(x\right)+\beta \mu N\right)$$

In eq 4, *V*° is the standard state volume and μ is the solvation free energy of a water molecule into water.^4^

For a cleaner notation later, here the Adams parameter B is defined (eq 5).^18,19^

5 $$B=\beta \mu +\ln\left(\frac{V_{GCMC}}{V^{{}^{\circ}}}\right)$$

When doing GCMC insertion/deletion with Metropolis-Hastings, the acceptance probabilities are.

Insertion *N* → *N* + 1

6 $$P_{insert}=\min\left[1,\frac{1}{N+1}\exp\left(B-\beta \Delta U\right)\right]$$

Deletion *N* → *N* – 1

7 $$P_{delete}=\min\left[1,N\exp\left(-B-\beta \Delta U\right)\right]$$

When we use nonequilibrium candidate Monte Carlo (NCMC)^20^ to enhance the acceptance rates, the interaction between the inserted/deleted water is gradually turned on/off, the energy difference is replaced by the work of the nonequilibrium processes (eqs 6, 7, 8, 9). However, during these processes, the water molecule might diffuse in or out of the selected volume. To circumvent this problem, first, if the inserted/deleted water molecule diffuses out of the selected volume, the move is rejected, as this move cannot be reversed (the deletion/insertion cannot start from the outside of the volume). Second, in the insertion, particle number *N* is counted at the end of the insertion, as the final *N* particles are indistinguishable. In the deletion, particle number *N* is counted at the start of the deletion, because the starting *N* particles are indistinguishable. We have the acceptance probabilities for GCMC performing with NCMC as.

Insertion *N*(*t*_0_) → *N*(*t*_end_)­

8 $$P_{insert}=\min\left[1,\frac{1}{N\left(t_{end}\right)}\exp\left(B-\beta w\right)\right]$$

Deletion *N*(*t*_0_) → *N*(*t*_end_)­

9 $$P_{delete}=\min\left[1,N\left(t_{0}\right)\exp\left(-B-\beta w\right)\right]$$

#### 2.1.2 Simulation Detail of GCMC

The whole sampling protocol alternated between 200 steps (0.8 ps) of NVT MD and 4 types of Monte Carlo moves.

(1) grand canonical insertion/deletion in the selected spherical region (GC_Sphere)
(2) grand canonical insertion/deletion in the whole box (GC_Box)
(3) terminal-flip
(4) replica exchange.

In the GC_Sphere one atom of the ligand was selected as the center of the sphere with a radius of 0.85 nm. In the GC equilibrium, 1 GC (either GC_Sphere or GC_Box with 50%:50% probability) was performed every 3.2 ps. In the GC production run, 1 GC (GC_Sphere/GC_Box = 20%:80%) was performed every 25.6 ps. The GC moves were performed in all λ-windows in parallel. In the thrombin test case when the terminal-flip Monte Carlo was applied, the terminal-flip (will be explained in Section 2.4) was performed every 3.2 ps in the equilibrium and every 51.2 ps in the production runs. The rest of the MC moves were replica exchanges (every 200 MD step, 0.8 ps).

The NCMC insertion/deletion was performed in 12.8 ps with 80 perturbation steps and 40 propagation (relaxation) steps. In deletion, the Coulomb interaction was turned off first followed by the vdW interaction and for the insertion the process was reversed.

### 2.2 Water-swap Monte Carlo (Water-swap MC

#### 2.2.1 Theory

As an alternative to grand-canonical insertion/deletion (μVT), we use a constant-particle-number water-swap Monte Carlo scheme in the *NPT* ensemble.^11^ In this approach, water molecules are exchanged between two spatial regions within the same periodic simulation box: (i) an active-site region and (ii) a bulk region.

We first define an active-site volume *V*_act_. The bulk region *V*_B_ is defined as the remaining allowed volume outside the active-site. At a given MC attempt, waters currently inside each region are counted as *N*_Act_ and *N*_B_, respectively.

The acceptance ratio is given by eqs 10 and 11.

In (bulk → active-site)

10 $$P_{in}=\min\left[1,\frac{N_{B}V_{act}}{\left(N_{act}+1\right)V_{B}}\exp\left(-\beta \Delta U\right)\right]$$

Out (active-site → bulk)

11 $$P_{out}=\min\left[1,\frac{N_{act}V_{B}}{\left(N_{B}+1\right)V_{act}}\exp\left(-\beta \Delta U\right)\right]$$

To improve the Monte Carlo acceptance rate, we use nonequilibrium candidate Monte Carlo (NCMC), in which interactions are switched gradually: the deleted water is alchemically decoupled while the inserted water is alchemically coupled. This staged switching relaxes steric clashes and can substantially increase acceptance relative to instantaneous moves (with the cost of more computation in each move).

During the nonequilibrium protocol, water molecules may diffuse across the active-site boundary. To preserve reversibility under our move definition, we reject any proposal in which the alchemically transformed water changes region (active-site ↔ bulk) during switching, because the corresponding reverse process cannot be performed. The acceptance ratio with NCMC is given by eqs 12 and 13.

In (bulk → active-site)

12 $$P_{in}=\min\left[1,\frac{N_{B}\left(t_{0}\right)V_{act}\left(t_{0}\right)}{N_{act}\left(t_{end}\right)V_{B}\left(t_{end}\right)}\exp\left(-\beta w\right)\right]$$

Out (active-site → bulk)

13 $$P_{out}=\min\left[1,\frac{N_{act}\left(t_{0}\right)V_{B}\left(t_{0}\right)}{N_{B}\left(t_{end}\right)V_{act}\left(t_{end}\right)}\exp\left(-\beta w\right)\right]$$

Note that in a **“In”** move, we use *N*_B_(*t*_0_) and *V*_act_(*t*_0_), because we randomly choose *N*_B_(*t*_0_) water to delete and insert the noninteracting water into the volume of *V*_act_(*t*_0_). *N*_act_(*t*_end_) and *V*_B_(*t*_end_) are used, because in the end of the NCMC process, the inserted water is the same as every other water in the active-site, and the noninteracting (deleted) water can be at any place in the volume of *V*_B_(*t*_end_). The **“Out”** move is counted in the same logic.

#### 2.2.2 Simulation Detail of Water-swap MC

The whole sampling protocol alternated between 200 steps (0.8 ps) of MD and 3 types of Monte Carlo Steps.

(1) swapping a water molecule in or out of the active-site (water-swap MC)
(2) terminal-flip
(3) replica exchange

In a water-swap MC move, one atom of the ligand was selected as the center of the sphere with a radius of approximately 0.85 nm, and the radius scaled linearly with the box vector. In the equilibration, the water-swap MC move was performed every 1.6 ps, and in the production run, the water-swap MC move was performed every 25.6 ps. The water-swap moves were performed in all λ-windows in parallel. In the thrombin test case when the terminal-flip Monte Carlo was applied, the terminal-flip (will be explained in Section 2.4) was performed every 3.2 ps in the equilibrium and every 51.2 ps in the production. The rest of the MC moves were replica exchanges (every 200 MD step, 0.8 ps).

The NCMC was performed in 12.8 ps with 80 perturbation steps and 40 propagation (relaxation) steps, and the inserted water and the deleted water were perturbed simultaneously.

### 2.3 REST2 (Replica Exchange with Solute Tempering 2)

REST2 is a Hamiltonian replica-exchange scheme in which only a selected subset of atoms (“hot” region, e.g., the ligand) is effectively tempered, while the rest of the system (solvent and “cold” atoms) remains unscaled.^21,22^ All replicas are simulated at the same thermostat temperature *T*_0_ but use different scaled Hamiltonians to reduce barriers in the hot region while maintaining reasonable overlap between replicas. The REST2 implemented in this work is given by eq 14.

14 $$U_{REST2}\left(x\right)={\left(\frac{T_{0}}{T_{M}}\right)}^{N_{hot}/N}U\left(x\right)$$

*T*_0_ is the reference temperature of the system, and *T*_M_ is the effective temperature of REST2. $\left(\frac{T_{0}}{T_{M}}\right)$ controls how much scaling down is set in REST2. *N* is the number of atoms in this interaction (for example, Coulomb is pairwise, *N* = 2). *N*_hot_ is the number of atoms in this interaction which is selected by REST2. The exponential term $\frac{N_{hot}}{N}$ controls that the hot–hot interaction is scaled by $\frac{T_{0}}{T_{M}}$ and the hot–cold interaction is scaled by $\sqrt{\frac{T_{0}}{T_{M}}}$ . Dihedral, vdW, Coulomb interactions are scaled and bond and angle interactions are untouched. *T*_0_ was set as 298.15 K and *T*_m_ increased from *T*_0_ in 1–8 replica to 870 K and decreased in 9–16 replica back to *T*_0_.

### 2.4 Terminal-Flip Monte Carlo (TFMC)

To improve sampling of terminal substituent orientations, we implemented a terminal-flip Monte Carlo (TFMC) move that proposes discrete rotations around selected terminal dihedral angles. The move is designed for terminal groups whose conformational interconversion is dominated by hindered torsional rotations, such as terminal phenyl rings. The move is performed within a standard Metropolis Monte Carlo framework, such that detailed balance is satisfied.^23^

In a TFMC move applied to a dihedral connecting a terminal phenyl ring, a new configuration is proposed by rotating the dihedral by 180°. For other types of dihedrals, the rotation direction is randomly chosen with equal probability, corresponding to positive or negative torsional rotations (sp^3^–sp^3^: ±120°, sp^2^(fixed)–sp^3^(rotate): ±60°). The new configuration is accepted with the following probability (eq 15)­

15 P=min[1,exp(−βΔU)]

After the Monte Carlo move is accepted, velocities for all atoms in the system are drawn from the Boltzmann distribution.

### 2.5 General Simulation Settings

All simulations were run in OpenMM using hydrogen mass repartitioning (HMR). Dynamics were propagated with a LangevinMiddleIntegrator (LFMiddle discretization) using a friction coefficient of 1 ps^–1^ and a time step of 4 fs.^24^ A Monte Carlo barostat with a reference pressure of 1 bar and an attempt frequency of 25 steps (100 fs) was used in NPT simulations. Nonbonded interactions used PME electrostatics and a 1.0 nm real-space cutoff. No switching function was applied to the vdW interactions. A long-range dispersion correction was enabled to approximate the contribution of vdW interactions beyond the cutoff. Beutler soft core potentials were used for vdW, no soft core was used for Coulomb.^25^ The system was modeled using Amber14SB^26^ with Tip3p^27^ water or Amber 19SB^28^ with OPC^29^ water. The ligand parameters were assigned with GAFF2 and am1-bcc charges;^30^ ligand topologies and charges were prepared using Antechamber and tleap (AmberTools). Atom mapping for alchemical transformations was generated using pmx.^31^ The normal dihedral potential involving dummy atoms were turned off, while the improper dihedral with dummy atoms were not turned off.

The ligand leg was simulated in the *NPT* ensemble using 16 λ-windows with 8 ns per window, and three independent repeats were performed. Prior to production, the λ schedule was optimized for each edge in the ligand leg. The resulting per-edge λ schedules were then reused for the corresponding protein-leg simulations. The optimization is explained in detail in Supporting Information. Relatively more sampling time was investigated in the ligand leg as we used the same ligand leg results for protein leg with GCMC and water-swap MC.

In the protein leg, we benchmarked GCMC and water-swap MC. For both protocols, the system was first equilibrated for 200 ps in the *NPT* ensemble with positional restraints applied to all protein and ligand heavy atoms (force constant 1000.0 kJ/(mol*nm^2^)). Production simulations were then initiated from the restrained *NPT* equilibrated state.

For GCMC replica exchange simulations, a unified box size is required for all the λ-windows. To preserve the density distribution (mean and standard deviation) of the final frame of the *NPT* equilibration, we scaled by 0.996 of the mean box size of the final frame in *NPT* equilibration, gave 16 λ-windows the same box size, and converted the reduced volume to dummy water molecules. With this, we initiated the GCMC replica exchange simulation with a reasonable density distribution in 16 λ-windows with a uniform box size. GCMC equilibration was run for 614 ps, followed by 15 ns of GCMC production. One independent protein-leg run was performed per edge.

For the water-swap MC protocol, simulations were also started from the same 200 ps restrained *NPT* equilibration, and equilibration and production lengths were matched to those used for GCMC (614 ps equilibration and 15 ns production).

The Δ*G* was derived from ΔΔ*G* using Cinnabar.^32^

In the insertion move of GCMC and water-swap MC, the newly inserted water molecules had random velocities drawing from the Boltzmann distribution of reference temperature with constraints considered.

## 3 Results and Discussion

### 3.1 Accurate RBFE

To evaluate the two water-enhanced sampling methods implemented in our GrandFEP package (GCMC and water-swap MC) we benchmarked both approaches on the published data set where water sampling is challenging.^4^ Across most systems, GCMC and water-swap MC achieved broadly comparable accuracy (Figure 1), with neither method consistently outperforming the other. Both delivered subkcal/mol RMSEs on several systems, including HSP90 (Woodhead et al.), Taf1(2), Urokinase.

**Figure 1:**
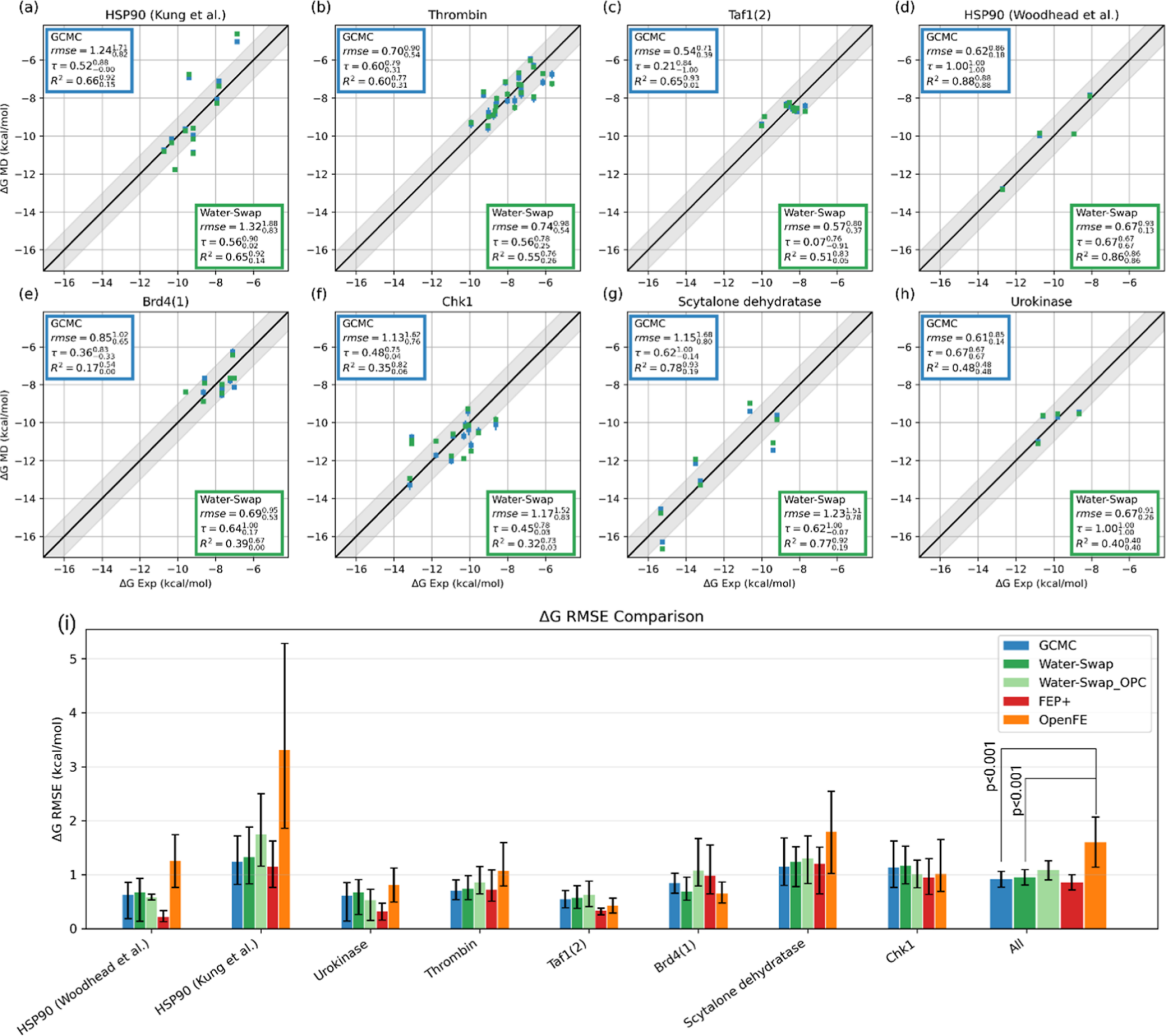
Accuracy and correlation between the experimental and predicted binding free energy (Δ*G*) for 8 systems using GCMC and water-swap MC. In the protocol of GCMC and water-swap MC, Amber14SB/GAFF2/TIP3P was used, and in the water-swap MC OPC protocol, Amber19SB/GAFF2/OPC was used. One repeat of the protein leg with 15 ns and three repeats of the ligand leg with 8 ns was simulated. The results of ligand leg with 1 repeat are shown in Figure S7 and Table S1. All the ΔΔ*G* and Δ*G* can be found in Supporting Information. (a) Scatter plot of each ligand in 8 systems. The gray regions are within 1 kcal/mol error. The error bar is the standard error. (b) Overall accuracy and correlation of different methods in 8 systems. The error bar of the RMSE, Kendall’s τ and *R* ^2^ are 95% confidence intervals that have been estimated by bootstrapping the ligands.

We further compare these results against two published baselines on the same benchmark set. FEP+ values were taken from the Ross et al. public benchmark data set (which provides a standardized mapping/simulation protocol and is distributed via Schrödinger’s public benchmark repository) and correspond to runs that employed GCMC within the FEP+ workflow.^12^ OpenFE values were taken from the recent large-scale OpenFE benchmarking study,^33^ which used Hamiltonian replica exchange RBFE protocols in NPT and can be taken as the baseline result without water enhanced sampling. GCMC and water-swap MC achieved weighted RMSEs of 0.92 and 0.95 kcal/mol respectively, compared to 0.86 kcal/mol for FEP+ and 1.60 kcal/mol for OpenFE. Both GCMC and water-swap MC significantly outperform OpenFE (*p* < 0.001, Figure 1i), demonstrating the clear benefit of explicit water sampling in RBFE calculations for systems with structured active-site water networks.

The same total number of sampling (FEP+:12 λ * 20 ns, GCMC/water-swap MC: 16 λ * 15 ns) and ligand mapping (network connecting ligands) was used in FEP+, GCMC and water-swap MC, and the results are close within the errorbar.^12^ This shows that our sampling engine with the enhanced sampling methods built in, provides similar sampling efficiency as FEP+. OpenFE yielded significantly poorer accuracy than FEP+, GCMC, and water-swap MC. A plausible contributor is the lack of explicit water-enhanced sampling, which is likely to matter for this benchmark because several targets involve kinetically trapped hydration states. Differences in total simulation time alone are unlikely to explain the gap. Although OpenFE used a smaller per-edge sampling budget (11 λ-windows × 5 ns × 3 repeats), the authors reported that the ΔΔ*G* estimates were largely converged within 5 ns per window and suggested that reducing to ∼4 ns would not substantially degrade accuracy. This supports the view that, in the absence of problem-specific enhanced sampling, simply running longer does not guarantee systematic error reduction; robust improvements require accelerating the relevant slow degrees of freedom (here, water occupancy and exchange), after which additional sampling can translate more directly into decreased uncertainty and bias.

We also checked the error of individual edges (Figure S2), the RMSE of the ΔΔ*G* for each system (Figure S3), the RMSE of the Δ*G* for each system (Figure S4). There was a slow trend of decreasing in the overall error even from the sampling of 15 to 20 ns. On this data set, simulations longer than 15 ns appear to provide limited additional improvement.

### 3.2 Hydrating Conserved Water Site

To assess the efficiency of water sampling, we monitored whether conserved hydration sites were reoccupied during the RBFE simulations. Both HSP90 and Brd4(1) feature four conserved hydration sites in the available crystal structures (Figure 2); however, three (HSP90) and two (Brd4(1)) of these waters were removed during system preparation because they sterically clash with some ligands. Figure 2 shows that both GCMC and water-swap MC rapidly rehydrate these sites, restoring the expected occupancy within ∼600 ps of equilibration across multiple perturbations (edges). Water-swap yielded relatively faster hydration equilibration, due to a higher number of trials per ns. A water-swap move was performed every 1.6 ps while a GC_Sphere move was performed on average every 6.4 ps.

**Figure 2:**
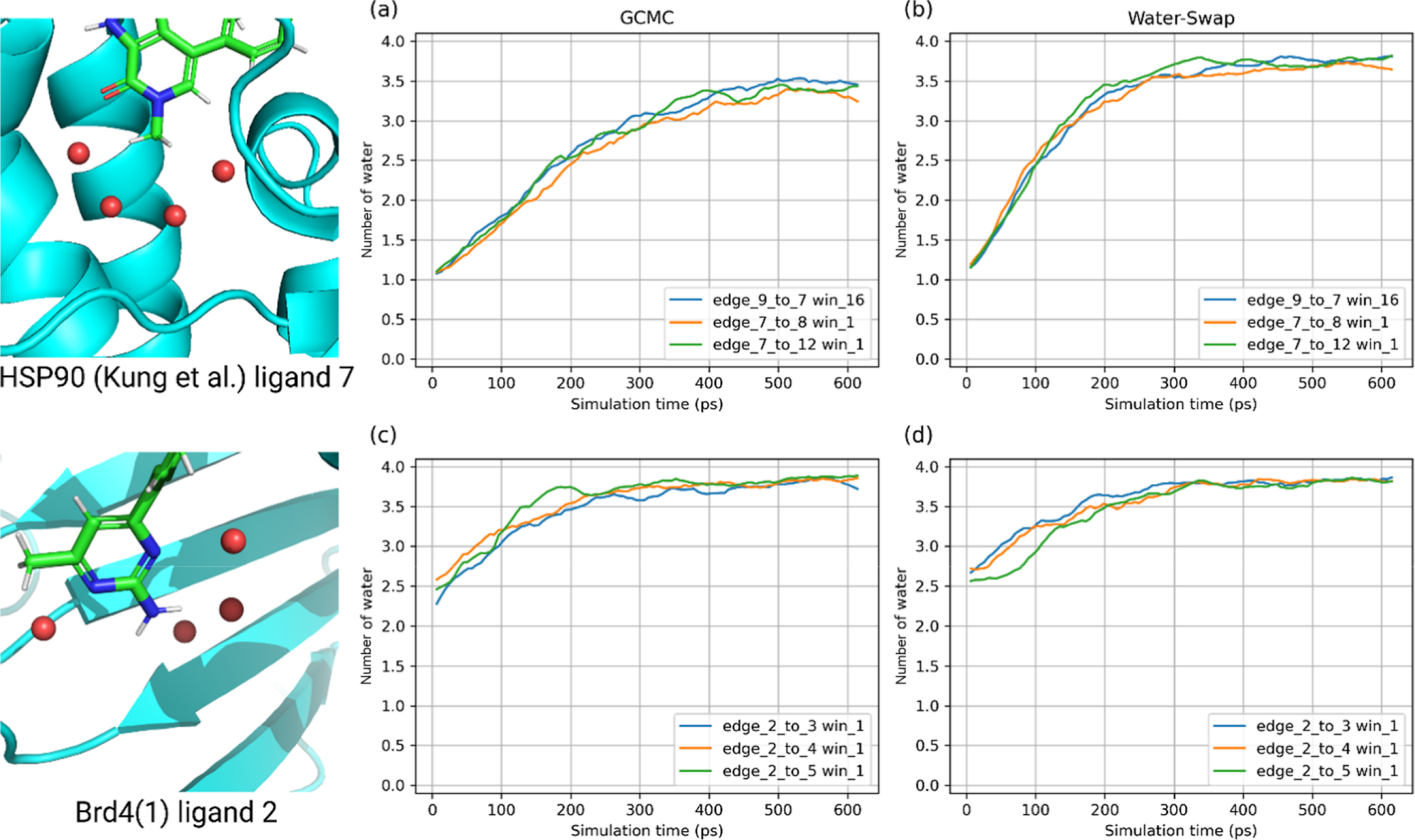
Rapid rehydration of conserved binding-site waters by GCMC and water-swap MC in RBFE (ΔΔ*G*) calculations. Conserved hydration sites (red spheres) in the binding pockets of HSP90 (first row, PDB: 3RLP) and Brd4(1) (second row, PDB: 3JVK) were rehydrated with two water sampling methods −GCMC (a,c) and water-swap MC (b,d). Each time series is averaged over 40 repeats.

Different cutoff radii for the active-site definition were also tested for the same rehydration equilibration. As we perform the same number of insertion/deletion trial every ns, the smaller the selected active-site, the faster the rehydration equilibration in the active-site. This anticorrelation between radius and speed of rehydration is shown in Figure S5. In practice, one should select the smallest active-site (radius) which can cover all the water binding sites. With all the radii tested, no significant difference of the Δ*G* RMSE was observed in the system tested (Figure S6).

### 3.3 Improving Conformation Sampling in Thrombin with Terminal-Flip Monte Carlo

Dihedral sampling can sometimes be a problem. Among the 8 systems tested in this work, thrombin poses a sampling challenge at the terminal phenyl ring. There are different substitutions on the phenyl ring and it is difficult to determine the pose of the asymmetrical phenyl ring. In the FEP+ paper, the authors calculated the ΔΔ*G* for both poses, which effectively doubled the computational cost.^4^ We tested two methods to tackle this sampling challenge, terminal-flip Monte Carlo and separate topology. In terminal-flip Monte Carlo, we still tried to map as many atoms as possible and added Monte Carlo steps to rotate the dihedral 180° (as explained in Section 2.4). In separate topology, the whole phenyl ring was not mapped between state A and state B, so that the dummy state of the phenyl ring could freely rotate and explore both orientations.^34^

Figure 3 compares binding free energy predictions across three strategies—Maximum Mapping, Terminal Flip MC, and Separate Topology—each evaluated with two water sampling schemes (GCMC and water-swap MC). Across all three approaches, predicted binding affinities correlate reasonably well with experimental values. However, ligands 6a and 6b, two of the strong binders in this series, show a notable qualitative improvement under Terminal Flip MC and Separate Topology relative to Maximum Mapping: both compounds shift visibly closer to the diagonal, suggesting that the torsional sampling correction specifically benefits these two cases.

**Figure 3:**
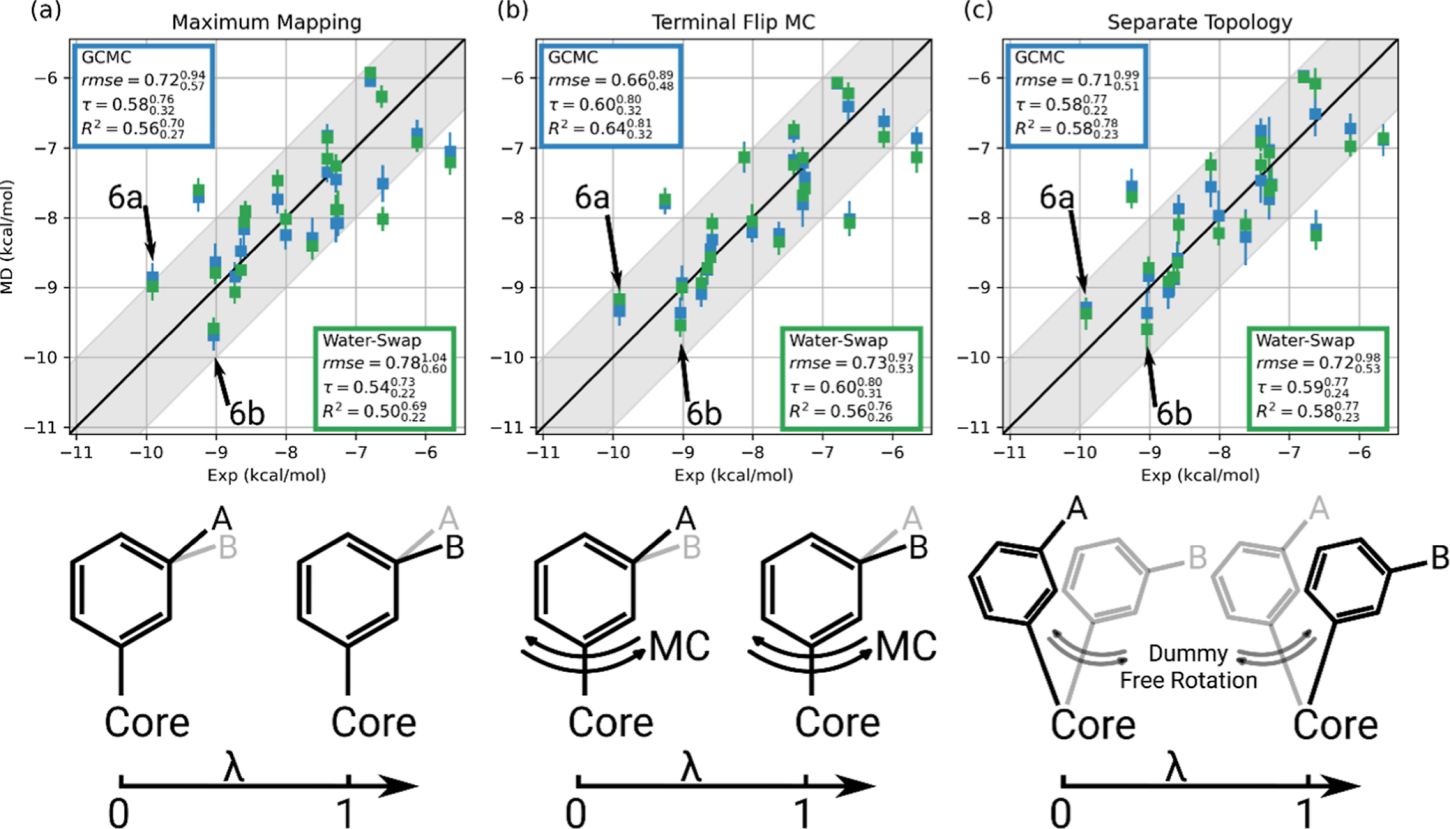
Impact of terminal-flip Monte Carlo and separate topology on thrombin RBFE calculations. (a) Maximum mapping, in which all common atoms (including the terminal phenyl ring) are mapped between states A and B. (b) Maximum mapping with terminal-flip Monte Carlo, where phenyl dihedral-flip moves are used to enhance sampling of terminal-ring orientations. (c) Separate-topology treatment of the phenyl ring, in which the ring is not mapped between A and B, allowing the decoupled dummy atoms (gray) to rotate freely and thereby accelerate sampling of phenyl dihedral reorientation during the alchemical transformation. Each ΔΔ*G* calculation is repeated 3 times, and each λ-window is simulated for 20 ns.

Without terminal-flip Monte Carlo or separate topology, the dihedral distributions were mostly trapped near the starting dihedral angle of −90°, and the ligand rarely crossed the torsional barrier to sample the alternative orientation (Figure 4). If the initial binding pose is difficult to determine, or if two orientations have similar free energies, an enhanced sampling method capable of crossing torsional barriers becomes necessary to obtain a reliable free energy estimate. Both terminal-flip Monte Carlo and separate topology efficiently sampled both orientations, yielding distributions with populations near both +90° and −90°. In ligands 6a and 6b, both orientations contributed meaningfully to the actual conformational ensemble in the bound state, meaning that a simulation trapped in a single orientation will systematically misestimate the free energy by neglecting the contribution of the alternative pose. The improvement in predicted binding affinity for these two ligands under the enhanced sampling methods is therefore a direct consequence of recovering this missing population.

**Figure 4:**
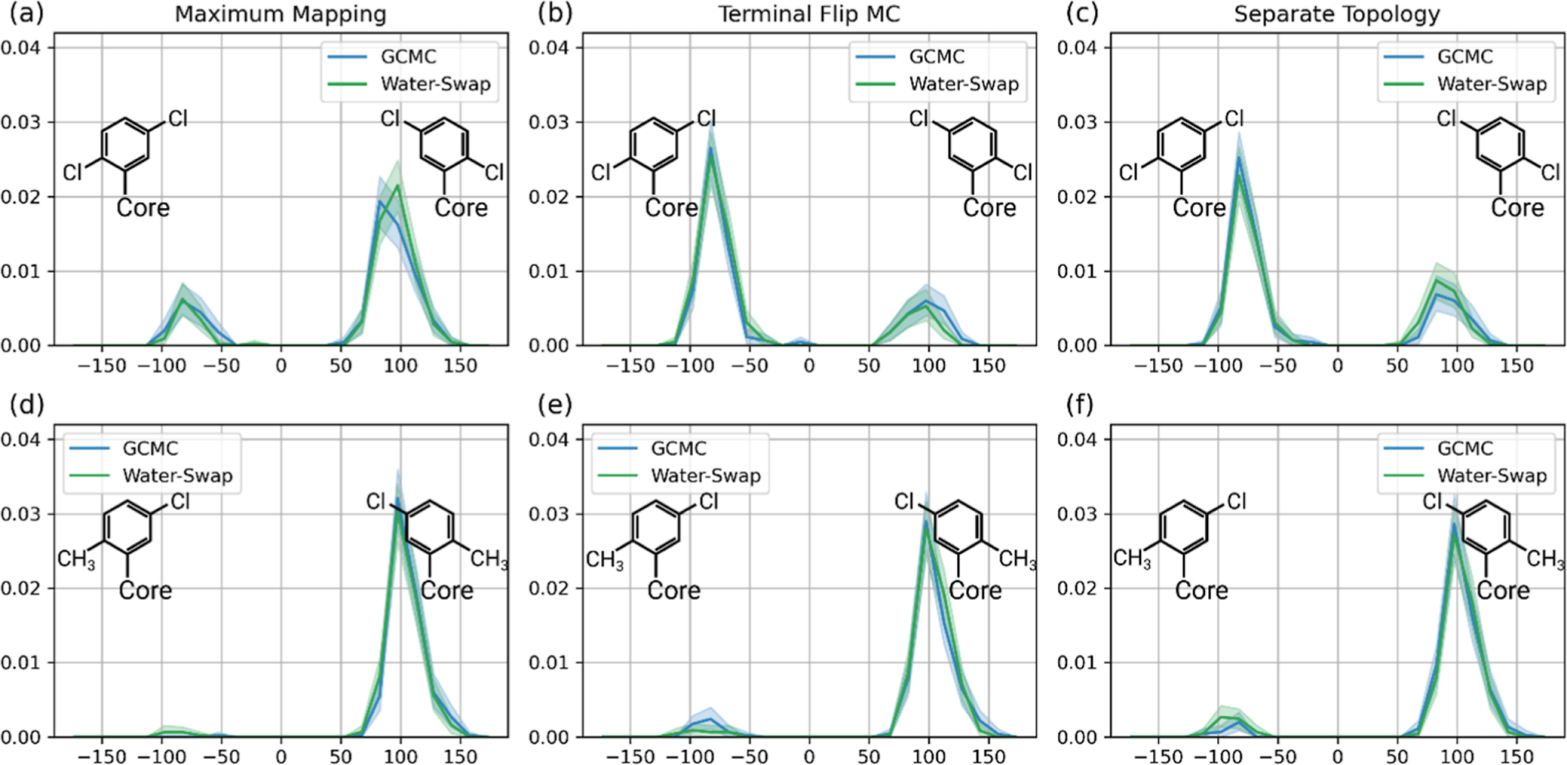
Effect of terminal-flip Monte Carlo on sampling of terminal phenyl dihedral angles in the thrombin test case. (a–c) Dihedral distribution of ligand 6a. (d–f) Dihedral distribution of ligand 6b.

### 3.4 Comparison between GCMC and Water-Swap MC

To validate that our GCMC protocol reproduces the correct bulk thermodynamic state, we compared the instantaneous density distributions obtained from GCMC sampling against a conventional NPT reference simulation (Figure 5). The replica-pooled density histogram is essentially indistinguishable from the NPT result in both mean and variance. This agreement indicates that the chosen chemical potential and insertion/deletion protocol are consistent with the target bulk water state and do not introduce a detectable bias in the equilibrium density distribution under these conditions.

**Figure 5:**
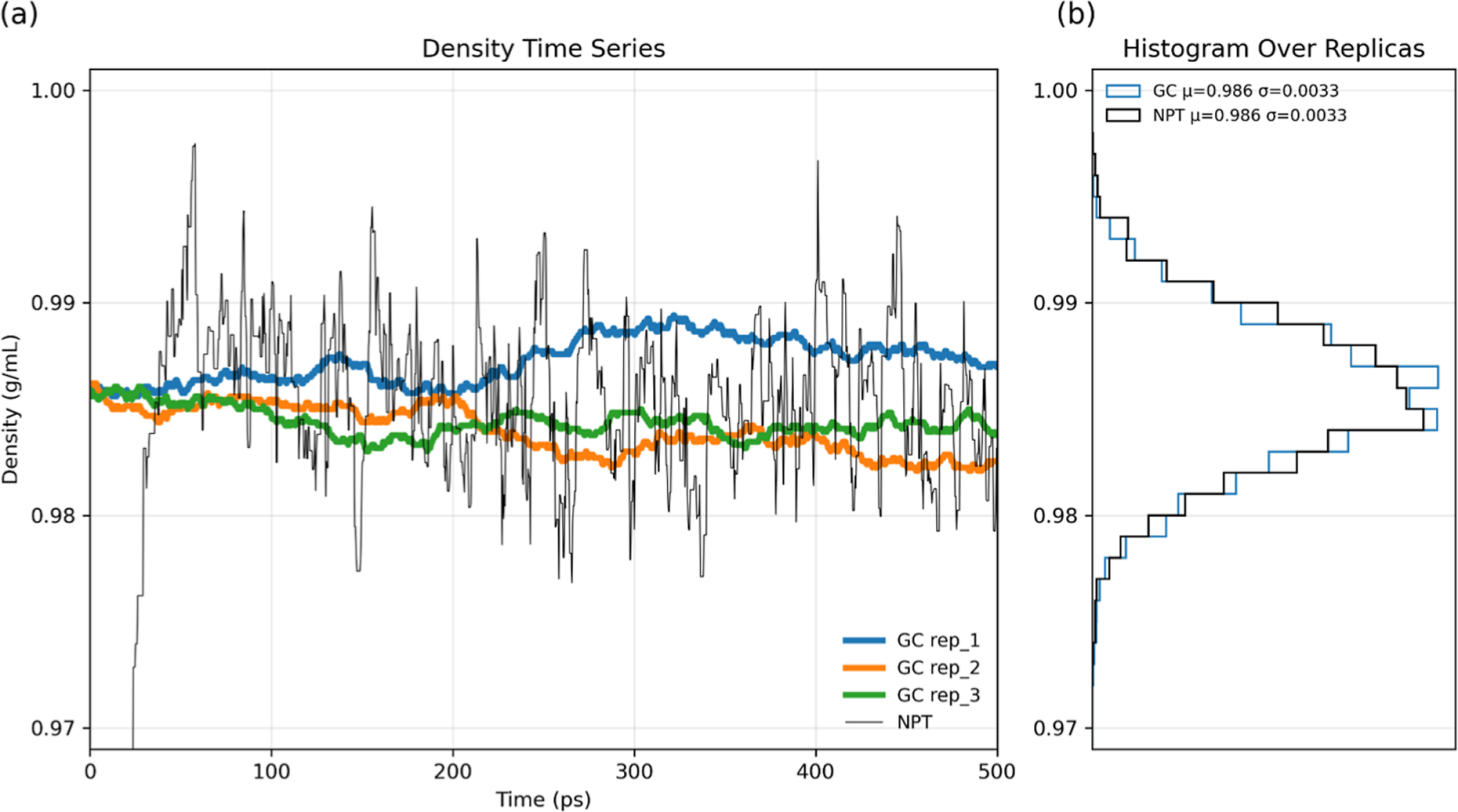
Bulk-water density fluctuations in GCMC and NPT simulations. (a) Time series of the instantaneous density from GCMC and NPT simulations; for visual clarity, the trace is shown as a zoom-in of the first 500 ps. (b) Density histograms from GCMC and NPT. GCMC simulations were run for 200 ns with 8 independent repeats, and NPT simulations were run for 15 ns with 5 independent repeats. The first 2% of each trajectory was discarded.

Although the equilibrium density statistics are correct, two practical considerations motivate the use of water-swap MC in production workflows. First, density (and effective volume) relaxation can be slow in GCMC. The NPT trajectory exhibits rapid density fluctuations driven by barostat-mediated volume changes (Figure 5), whereas the GCMC replicates show slower drift and longer correlation times. This behavior is expected because, in GCMC, changes in the number of particles (and therefore bulk density) occur through discrete insertion/deletion MC steps; consequently, even when the stationary density distribution is correct, the dynamics of density equilibration can be slower—and therefore more costly to decorrelate—than under an NPT barostat. In contrast, water-swap MC preserves a fixed particle number and primarily accelerates local hydration exchange in a predefined region, which reduces sensitivity to slow global density relaxation.

Second, GCMC is not straightforward to deploy in semi-isotropic ensembles, such as membrane simulations, where fluctuations in box dimensions are anisotropic and tightly coupled to surface tension, lipid packing, and bilayer thickness. In such settings, coupling GCMC insertions/deletions to the correct thermodynamic ensemble and calibration can be nontrivial, and extending number-fluctuation moves to additional components (e.g., lipids) would be prohibitively expensive in practice. By design, water-swap MC is compatible with standard barostats (including semi-isotropic coupling), since it exchanges water between bulk and a target region without changing the total number of molecules. This makes water-swap MC a practical method for membrane-associated targets while still enabling rapid equilibration of buried hydration sites. Considering the small computational overhead in water-swap MC compared to GCMC (Figure S1), we recommend water-swap MC for general simulations.

## 4 Conclusion

In this work we introduced GrandFEP, an open-source RBFE sampling engine built on OpenMM that targets key convergence bottlenecks in lead-optimization free energy calculations. Beyond a standard alchemical workflow, GrandFEP natively integrates enhanced sampling moves and replica-based acceleration so that kinetically trapped degrees of freedom—most notably binding-site hydration states and slow ligand conformations—can equilibrate during ΔΔ*G* estimation rather than being fixed by initial modeling choices.

GrandFEP provides two complementary approaches for hydration sampling. GCMC enables explicit insertion/deletion of water molecules, analogous in spirit to the GCMC-enabled workflows widely used in commercial FEP pipelines. Water-swap MC offers an alternative route to equilibrating pocket hydration by exchanging/relocating water without relying on grand-canonical number fluctuations, which is more efficient and capable of simulating semi-isotropic membrane systems. In addition, GrandFEP implements REST2 to accelerate ligand conformational sampling, and includes terminal-flip Monte Carlo moves to efficiently sample discrete terminal-group reorientations. Together, these capabilities provide a unified and extensible framework for robust RBFE calculations in challenging systems and facilitate transparent community benchmarking and further method development.

## Supplementary Material







## Data Availability

All simulation code and analysis scripts are publicly available on GitHub at: https://github.com/deGrootLab/GrandFEP.

## Notes

All the calculated ΔΔ*G* and Δ*G* are provided in ddG_dG_data.zip (ZIP)
Additional extended figures are provided in GrandFEP_GCMC_RBFE_SI.pdf (PDF)
All the starting structures are provided in structures.zip (ZIP)

## References

[1] Dunitz J. D. (1994). The Entropic
Cost of Bound Water in Crystals and Biomolecules. Science.

[2] Lu Y., Wang R., Yang C.-Y., Wang S. (2007). Analysis of Ligand-Bound
Water Molecules in High-Resolution Crystal Structures of Protein–Ligand
Complexes. J. Chem. Inf. Model..

[3] Denisov V. P., Halle B., Peters J., Hoerlein H. D. (1995). Residence Times
of the Buried Water Molecules in Bovine Pancreatic Trypsin Inhibitor
and Its G36S Mutant. Biochemistry.

[4] Ross G. A., Russell E., Deng Y., Lu C., Harder E. D., Abel R., Wang L. (2020). Enhancing Water Sampling
in Free
Energy Calculations with Grand Canonical Monte Carlo. J. Chem. Theory Comput..

[5] Samways M. L., Bruce Macdonald H. E., Essex J. W. (2020). Grand: A Python
Module for Grand
Canonical Water Sampling in OpenMM. J. Chem.
Inf. Model..

[6] Melling O. J., Samways M. L., Ge Y., Mobley D. L., Essex J. W. (2023). Enhanced
Grand Canonical Sampling of Occluded Water Sites Using Nonequilibrium
Candidate Monte Carlo. J. Chem. Theory Comput..

[7] Bergazin T. D., Ben-Shalom I. Y., Lim N. M., Gill S. C., Gilson M. K., Mobley D. L. (2021). Enhancing
Water Sampling of Buried Binding Sites Using
Nonequilibrium Candidate Monte Carlo. J. Comput.
Aided Mol. Des..

[8] Ben-Shalom I. Y., Lin C., Radak B. K., Sherman W., Gilson M. K. (2021). Fast Equilibration
of Water between Buried Sites and the Bulk by Molecular Dynamics with
Parallel Monte Carlo Water Moves on Graphical Processing Units. J. Chem. Theory Comput..

[9] Ben-Shalom I. Y., Lin Z., Radak B. K., Lin C., Sherman W., Gilson M. K. (2020). Accounting
for the Central Role of Interfacial Water in Protein–Ligand
Binding Free Energy Calculations. J. Chem. Theory
Comput..

[10] Ben-Shalom I. Y., Lin C., Kurtzman T., Walker R. C., Gilson M. K. (2019). Simulating Water
Exchange to Buried Binding Sites. J. Chem. Theory
Comput..

[11] Ding, Y. ; Wang, X. ; Zou, R. ; Zheng, H. Improving Binding Free Energy Predictions with Swap Monte Carlo for Water Sampling. July 15, 2025. 10.1021/acs.jctc.6c00178 42579379

[12] Ross G. A., Lu C., Scarabelli G., Albanese S. K., Houang E., Abel R., Harder E. D., Wang L. (2023). The Maximal and Current Accuracy
of Rigorous Protein-Ligand Binding Free Energy Calculations. Commun. Chem..

[13] Gill S. C., Lim N. M., Grinaway P. B., Rustenburg A. S., Fass J., Ross G. A., Chodera J. D., Mobley D. L. (2018). Binding
Modes of Ligands Using Enhanced Sampling (BLUES): Rapid Decorrelation
of Ligand Binding Modes via Nonequilibrium Candidate Monte Carlo. J. Phys. Chem. B.

[14] Wagle S., Bayly C. I., Mobley D. L. (2025). Advancing Binding
Affinity Calculations:
A Non-Equilibrium Simulations Approach for Calculation of Relative
Binding Free Energies in Systems with Trapped Waters. J. Chem. Theory Comput..

[15] Gracia
Carmona O., Gillhofer M., Tomasiak L., De Ruiter A., Oostenbrink C. (2023). Accelerated Enveloping Distribution Sampling to Probe
the Presence of Water Molecules. J. Chem. Theory
Comput..

[16] Eastman P., Galvelis R., Peláez R. P., Abreu C. R. A., Farr S. E., Gallicchio E., Gorenko A., Henry M. M., Hu F., Huang J., Krämer A., Michel J., Mitchell J. A., Pande V. S., Rodrigues J. P., Rodriguez-Guerra J., Simmonett A. C., Singh S., Swails J., Turner P., Wang Y., Zhang I., Chodera J. D., De Fabritiis G., Markland T. E. (2024). OpenMM 8: Molecular Dynamics Simulation with Machine
Learning Potentials. J. Phys. Chem. B.

[17] Frenkel, D. ; Smit, B. Monte Carlo Simulations in Various Ensembles. In Understanding Molecular Simulation; Elsevier, 2002; pp 111–137.

[18] Adams D. J. (1974). Chemical
Potential of Hard-Sphere Fluids by Monte Carlo Methods. Mol. Phys..

[19] Adams D. J. (1975). Grand Canonical
Ensemble Monte Carlo for a Lennard-Jones Fluid. Mol. Phys..

[20] Nilmeier J. P., Crooks G. E., Minh D. D. L., Chodera J. D. (2011). Nonequilibrium Candidate
Monte Carlo Is an Efficient Tool for Equilibrium Simulation. Proc. Natl. Acad. Sci. U. S. A..

[21] Liu P., Kim B., Friesner R. A., Berne B. J. (2005). Replica Exchange
with Solute Tempering:
A Method for Sampling Biological Systems in Explicit Water. Proc. Natl. Acad. Sci. U. S. A..

[22] Wang L., Friesner R. A., Berne B. J. (2011). Correction to “Replica
Exchange
with Solute Scaling: A More Efficient Version of Replica Exchange
with Solute Tempering (REST2).”. J. Phys.
Chem. B.

[23] Wang X., Ding Y., Zou R., Qu T., Zheng H. (2026). Terminal-Flip
Monte Carlo: Accelerating the Convergence of Molecular Dynamics and
Alchemical Free Energy Calculations. J. Chem.
Theory Comput..

[24] Zhang Z., Liu X., Yan K., Tuckerman M. E., Liu J. (2019). Unified Efficient Thermostat
Scheme for the Canonical Ensemble with Holonomic or Isokinetic Constraints
via Molecular Dynamics. J. Phys. Chem. A.

[25] Beutler T. C., Mark A. E., Van Schaik R. C., Gerber P. R., Van Gunsteren W. F. (1994). Avoiding
Singularities and Numerical Instabilities in Free Energy Calculations
Based on Molecular Simulations. Chem. Phys.
Lett..

[26] Maier J. A., Martinez C., Kasavajhala K., Wickstrom L., Hauser K. E., Simmerling C. (2015). ff14SB: Improving
the Accuracy of
Protein Side Chain and Backbone Parameters from ff99SB. J. Chem. Theory Comput..

[27] MacKerell A. D., Bashford D., Bellott M., Dunbrack R. L., Evanseck J. D., Field M. J., Fischer S., Gao J., Guo H., Ha S., Joseph-McCarthy D., Kuchnir L., Kuczera K., Lau F. T. K., Mattos C., Michnick S., Ngo T., Nguyen D. T., Prodhom B., Reiher W. E., Roux B., Schlenkrich M., Smith J. C., Stote R., Straub J., Watanabe M., Wiórkiewicz-Kuczera J., Yin D., Karplus M. (1998). All-Atom Empirical Potential for Molecular Modeling
and Dynamics Studies of Proteins. J. Phys. Chem.
B.

[28] Tian C., Kasavajhala K., Belfon K. A. A., Raguette L., Huang H., Migues A. N., Bickel J., Wang Y., Pincay J., Wu Q., Simmerling C. (2020). ff19SB: Amino-Acid-Specific Protein Backbone Parameters
Trained against Quantum Mechanics Energy Surfaces in Solution. J. Chem. Theory Comput..

[29] Izadi S., Anandakrishnan R., Onufriev A. V. (2014). Building Water Models:
A Different
Approach. J. Phys. Chem. Lett..

[30] Wang J., Wolf R. M., Caldwell J. W., Kollman P. A., Case D. A. (2004). Development
and Testing of a General Amber Force Field. J. Comput. Chem..

[31] Gapsys V., Michielssens S., Seeliger D., de Groot B. L. (2015). Pmx: Automated Protein
Structure and Topology Generation for Alchemical Perturbations. J. Comput. Chem..

[32] Hahn D., Bayly C., Boby M. L., Bruce Macdonald H., Chodera J., Gapsys V., Mey A., Mobley D., Perez Benito L., Schindler C., Tresadern G., Warren G. (2022). Best Practices for Constructing, Preparing, and Evaluating
Protein-Ligand Binding Affinity Benchmarks [Article v1.0]. Living J. Comput. Mol. Sci..

[33] Baumann, H. M. ; Horton, J. T. ; Henry, M. M. ; Travitz, A. ; Ries, B. ; Gowers, R. J. ; Swenson, D. W. H. ; Pulido, I. ; Rufa, D. ; Dotson, D. L. ; Bansal, N. ; Bluck, J. P. ; Broughton, H. ; Campbell, K. ; Cao, L. ; Frieg, B. ; Gapsys, V. ; Göddeke, H. ; Klähn, M. ; Lakkaraju, S. K. ; Linker, S. M. ; Löhr, T. ; Magarkar, A. ; Pérez-Conesa, S. ; Purkey, H. E. ; Saribekyan, H. ; Scheen, J. ; Schindler, C. E. M. ; Steinbrecher, T. ; Stern, C. D. ; Suriana, P. ; Swope, W. C. ; Tresadern, G. ; Tsidilkovski, L. ; Wei, B. ; Williams, A. H. ; Wu, Y. ; Zhang, I. ; Chodera, J. D. ; Eastwood, J. R. B. ; Mobley, D. L. ; Alibay, I. Large-Scale Collaborative Assessment of Binding Free Energy Calculations for Drug Discovery Using OpenFE. December 2025, 18. 10.26434/chemrxiv-2025-7sthd. PMC13555741 42165748

[34] Baumann H. M., Dybeck E., McClendon C. L., Pickard F. C., Gapsys V., Pérez-Benito L., Hahn D. F., Tresadern G., Mathiowetz A. M., Mobley D. L. (2023). Broadening the Scope of Binding Free
Energy Calculations Using a Separated Topologies Approach. J. Chem. Theory Comput..

