# Supplementary material for: Enhancing Relative Binding Free Energy Calculation with Grand Canonical Monte Carlo, Water-swap Monte Carlo, Terminal-flip Monte Carlo and Replica Exchange Solute Tempering
Source: J Chem Theory Comput. 2026 Jun 15;22(13):6709–17. doi: 10.1021/acs.jctc.6c00593 (PMC13374029; doi:10.1021/acs.jctc.6c00593)
Supplement: Supplementary file 2 [file ct6c00593_si_002.pdf]

Enhancing Relative Binding Free Energy Calculation with Grand Canonical Monte Carlo, Water-Swap Monte Carlo, Terminal-Flip Monte Carlo and Replica Exchange Solute Tempering

*Chenggong Hui[1], Bert L. de Groot[1]**

[1]Computational Biomolecular Dynamics Group, Max Planck Institute for Multidisciplinary Sciences, Am Fassberg 11, 37077 Göttingen.

bgroot@gwdg.de

1. Simulation efficiency and computational overhead

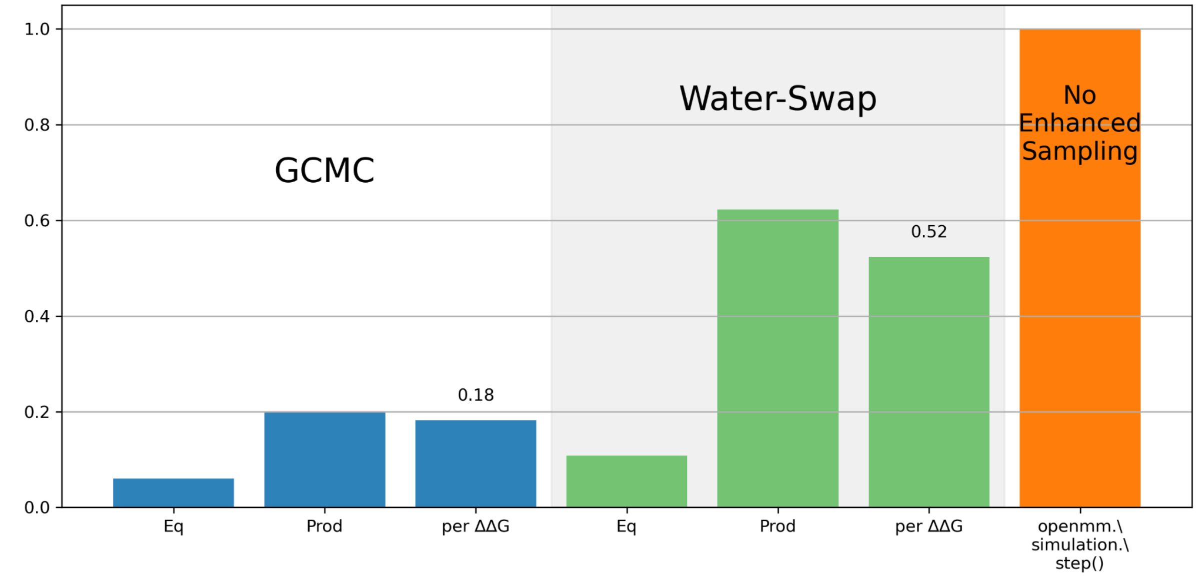

Figure S1. Relative simulation speed of GCMC and WaterMC at equilibrium phase and production run phase. The native openmm.simulation.step() without any enhanced sampling was chosen as the reference speed. The simulation length here only counts the equilibrium trajectory, not the nonequilibrium candidate Monte Carlo trajectory. The test was performed on a Ryzen 7900X CPU with a 3090 GPU.

Figure S1 quantifies the simulation overhead introduced by water sampling in a representative FEP simulation setting. The equilibration and production run protocols are described in sections 2.1.2 and 2.2.2. During equilibration, the higher number of insertion/deletion Monte Carlo trials resulted in substantially reduced simulation speed for both GCMC and WaterMC relative to the unmodified OpenMM baseline. Note that these speeds were measured as the equilibrium trajectory only, excluding the non-equilibrium candidate Monte Carlo trajectory. In GCMC, the presence of ghost water molecules in the simulation box increases the frequency of neighbour list updates, reducing simulation throughput. Weighted by the respective durations of equilibration (614 ps) and production (15 ns), the overall relative simulation speed per ΔΔG calculation was 0.18 for GCMC and 0.52 for WaterMC.

2. Lambda Path Optimization

The goal of the optimization is to redistribute the lambda windows along the alchemical pathway such that each neighbouring pair of windows contributes equally to the total error. This produces a more statistically efficient spacing than a uniform grid.

The algorithm operates iteratively. At each optimization step, it takes the current lambda schedule $\lambda = \{\lambda_1, \lambda_2, \ldots, \lambda_N\}$ and a vector of pairwise errors $\{e_{0,1}, e_{1,2}, \ldots, e_{N-1,N}\}$, where $e_{i,i+1}$ is the estimated error between windows λ_i and λ_{i+1} (e.g. from MBAR).

Update rule

For each interval, the current step size is:

$$S_{i,i+1} = \lambda_{i+1} - \lambda_i$$

The new target step sizes are chosen proportional to the ratio $S_{i,i+1}/e_{i,i+1}$. Intuitively, if an interval is wide ($S_{i,i+1}$ large) but has low error ($e_{i,i+1}$ small), it can afford to be wider; conversely, a narrow interval with large error should be compressed. Normalising these targets to sum to 1 gives a new set of fractional lengths, from which a new lambda schedule is reconstructed by cumulative summation.

Application

The optimization was performed using the ligand-in-water leg, where sampling is cheaper and convergence is faster. The resulting optimized lambda schedule was then applied unchanged to the ligand-in-protein leg, under the assumption that the regions of the alchemical pathway requiring finer resolution are largely determined by the shape changes of the ligand and are therefore transferable between the two environments.

3. Convergence check

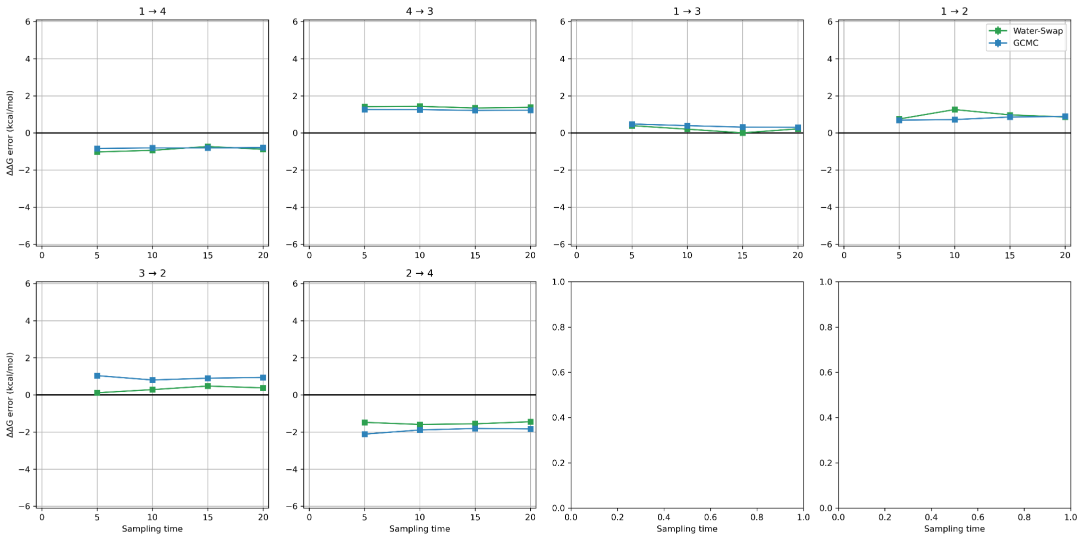

Figure S2.1. Error in the calculated ΔΔG for each edge in the HSP90 (Woodhead et al.) simulated with Amber14SB/GAFF2/TIP3P.

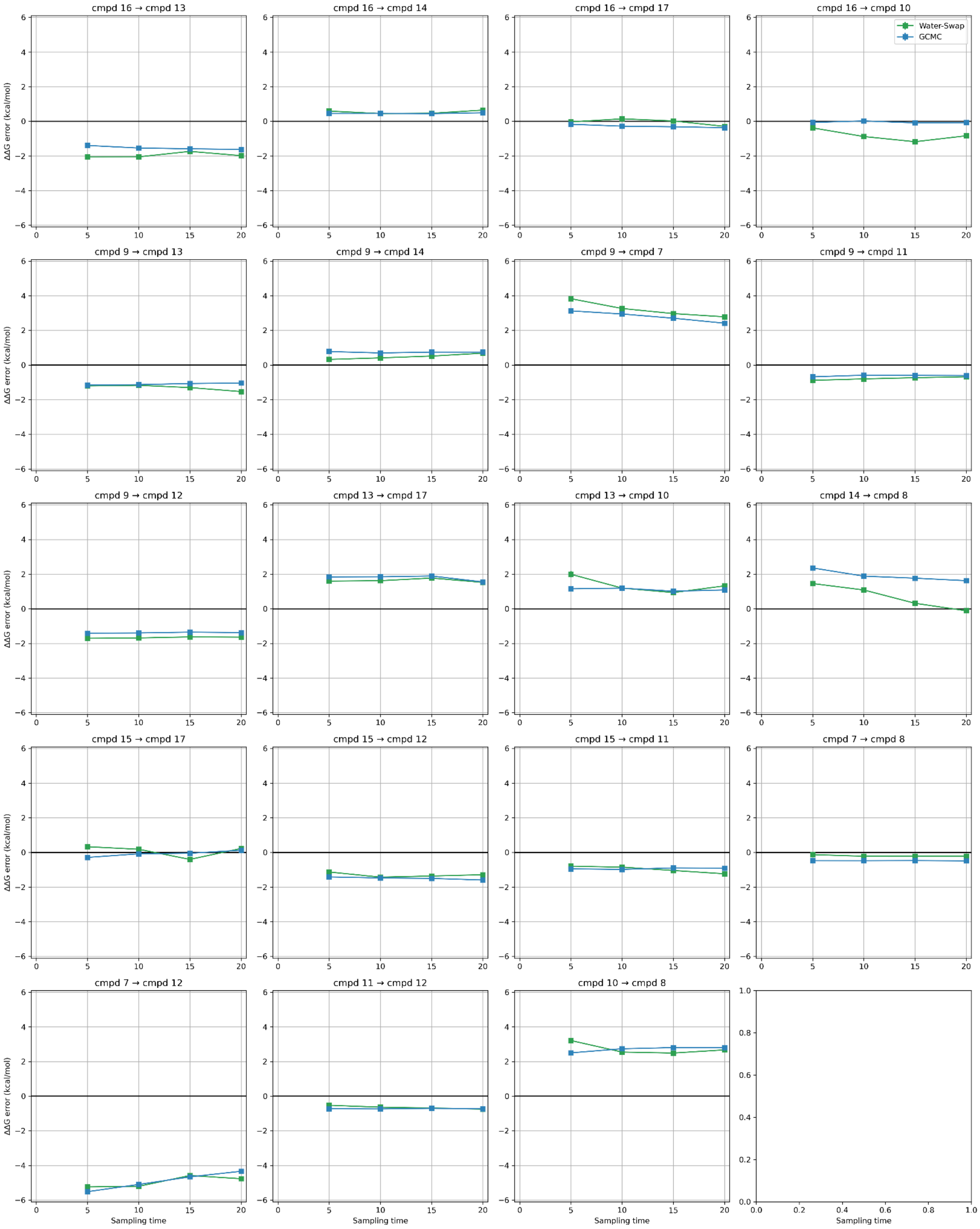

Figure S2.2. Error in the calculated ΔΔG for each edge in the HSP90 (Kung et al.) simulated with Amber14SB/GAFF2/TIP3P.

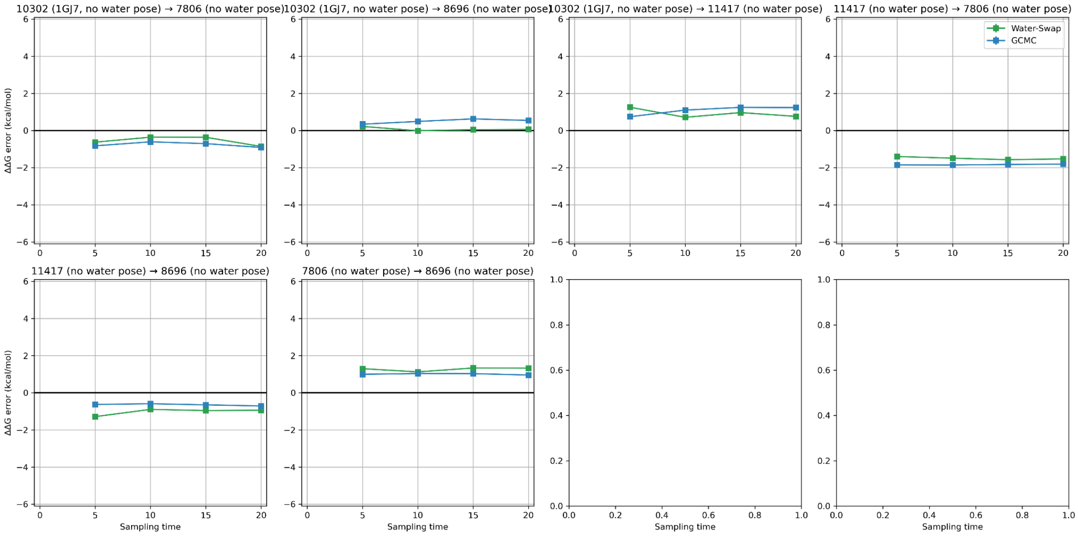

Figure S2.3. Error in the calculated ΔΔG for each edge in the Urokinase simulated with Amber14SB/GAFF2/TIP3P.

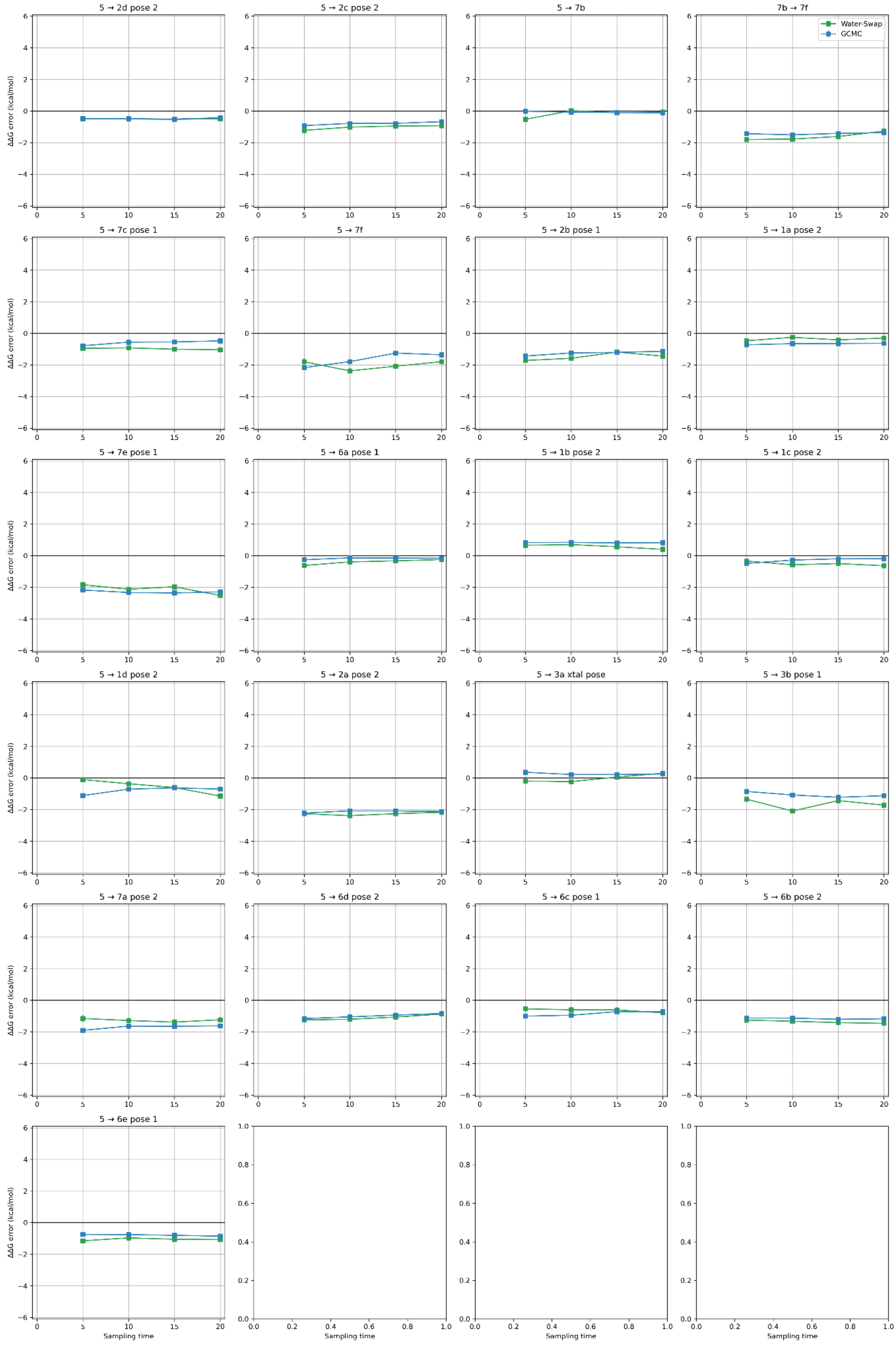

Figure S2.4. Error in the calculated ΔΔG for each edge in the Thrombin simulated with Amber14SB/GAFF2/TIP3P.

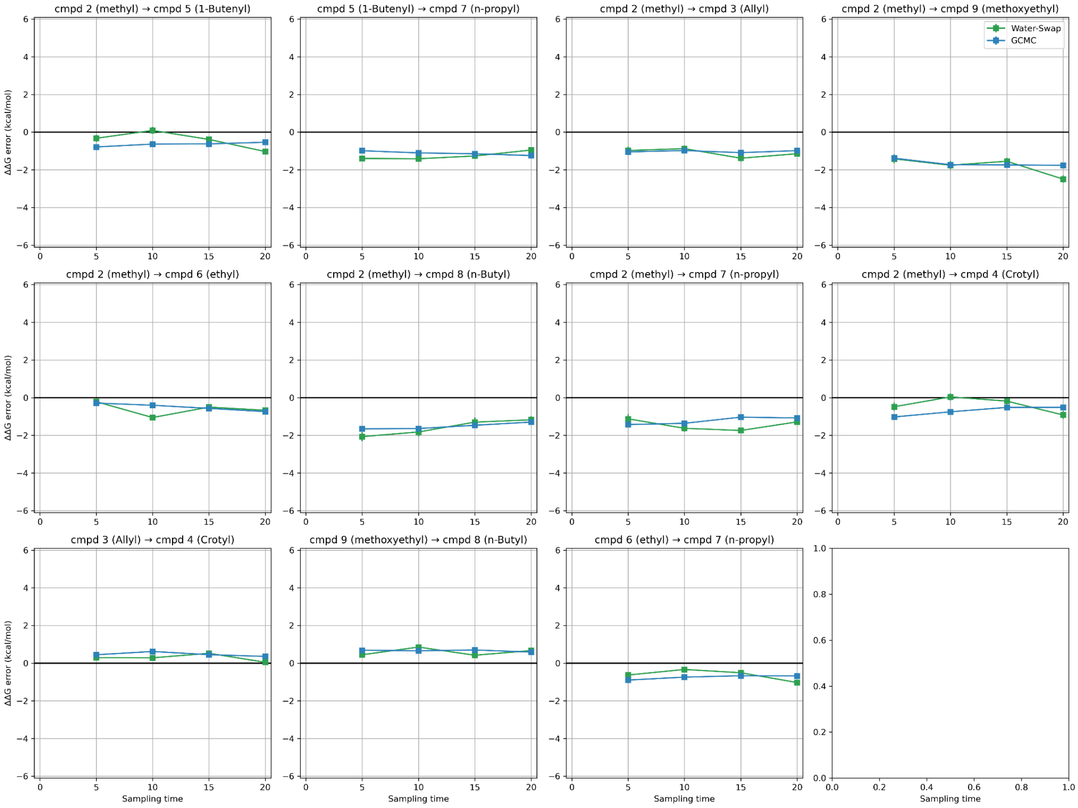

Figure S2.5. Error in the calculated ΔΔG for each edge in the Taf1(2) simulated with Amber14SB/GAFF2/TIP3P.

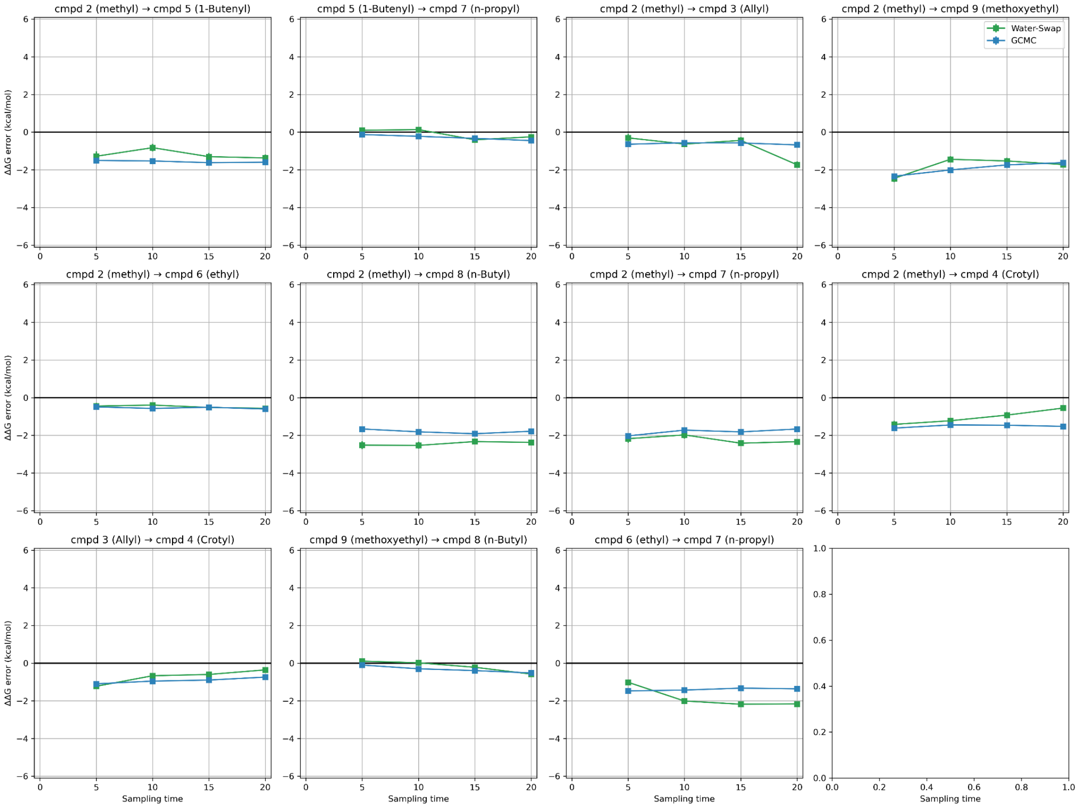

Figure S2.6. Error in the calculated ΔΔG for each edge in the BRD4(1) simulated with Amber14SB/GAFF2/TIP3P.

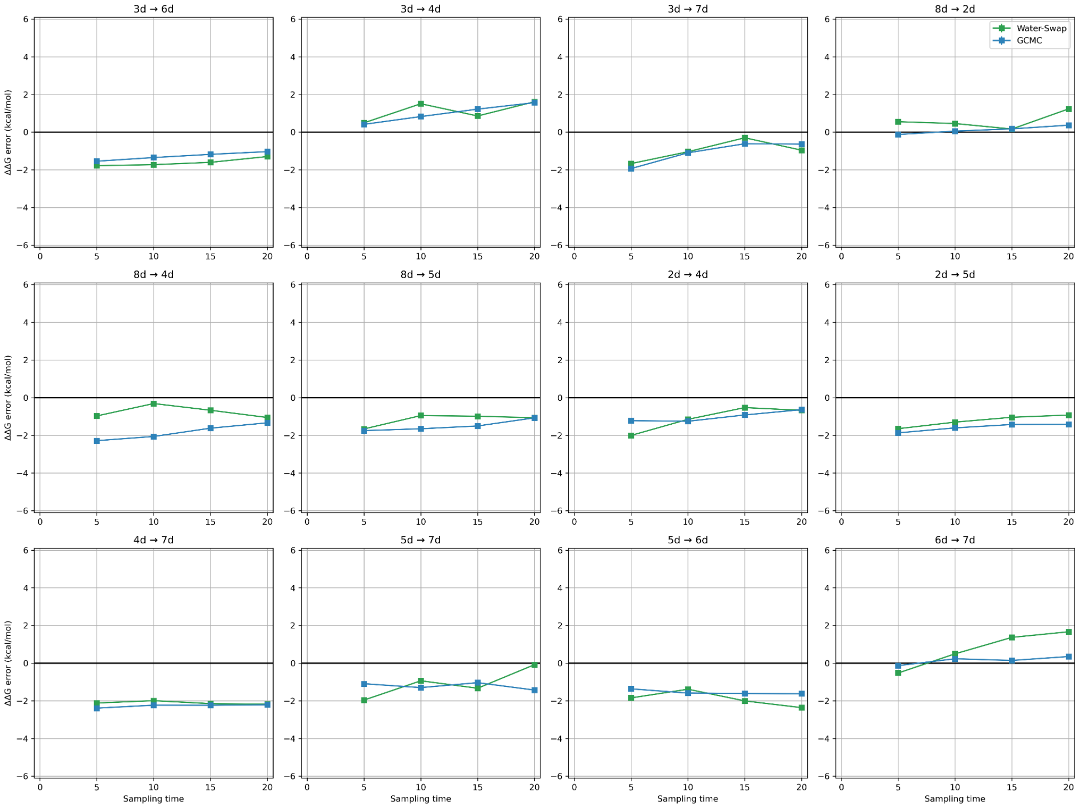

Figure S2.7. Error in the calculated ΔΔG for each edge in the Scytalone dehydratase simulated with Amber14SB/GAFF2/TIP3P.

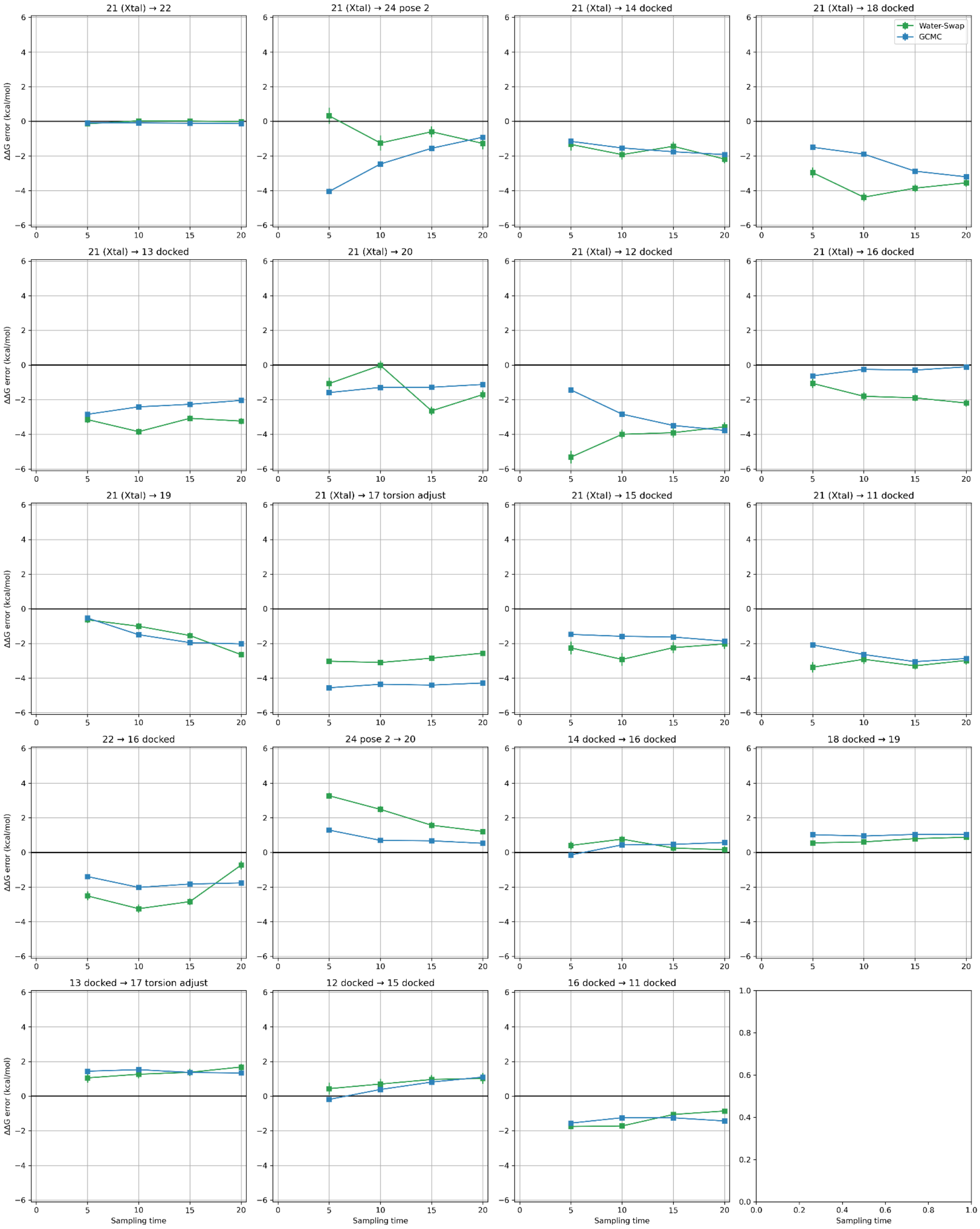

Figure S2.8. Error in the calculated ΔΔG for each edge in the Chk1 simulated with Amber14SB/GAFF2/TIP3P.

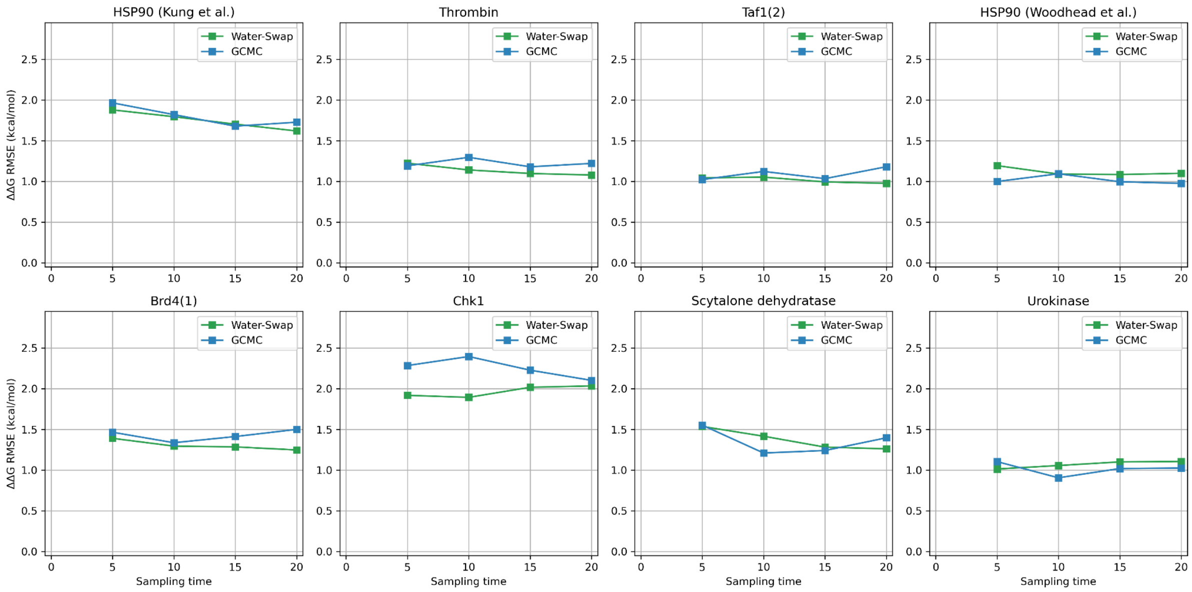

Figure S3.1. RMSE of ΔΔG for 8 systems simulated with Amber14SB/GAFF2/TIP3P

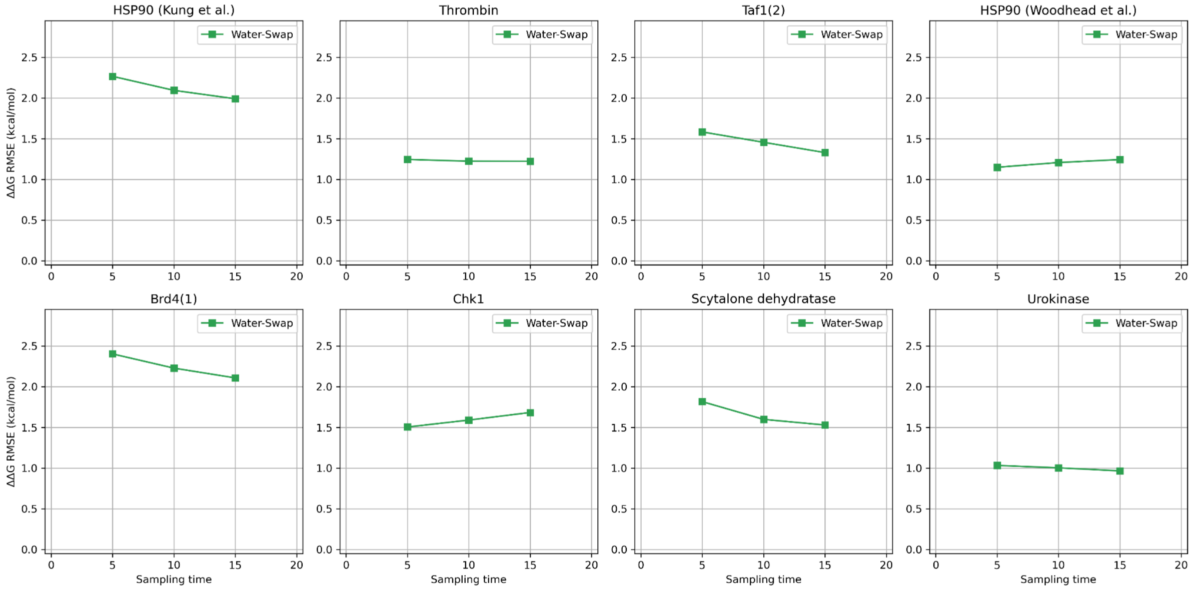

Figure S3.2. RMSE of ΔΔG for 8 systems simulated with Amber19SB/GAFF2/OPC

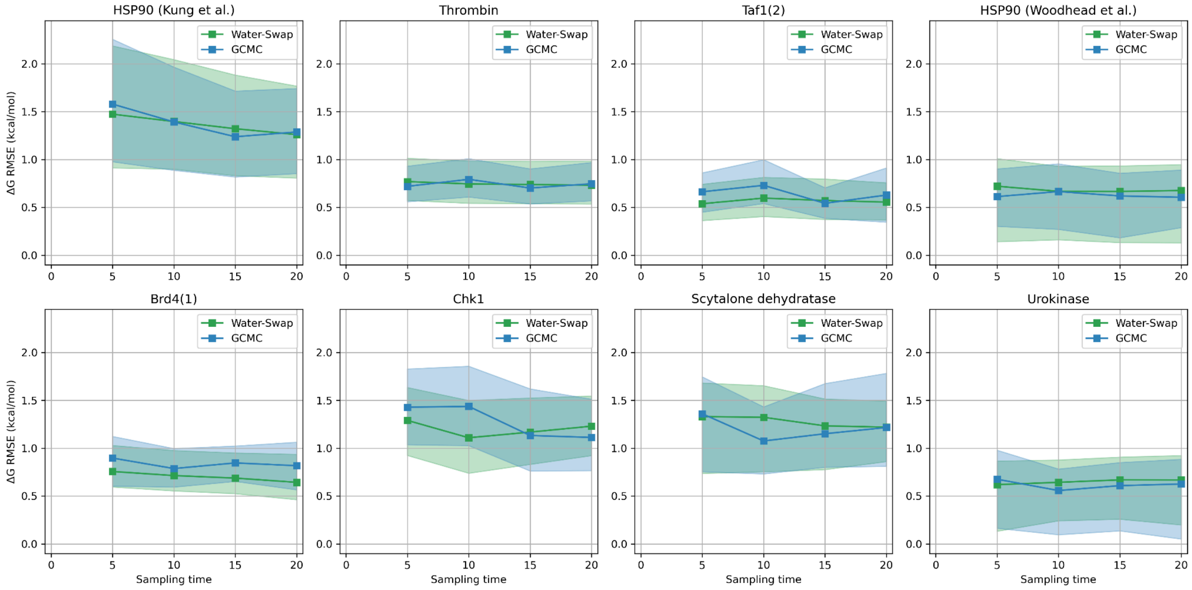

Figure S4.1. RMSE of ΔG for 8 systems simulated with Amber14SB/GAFF2/TIP3P

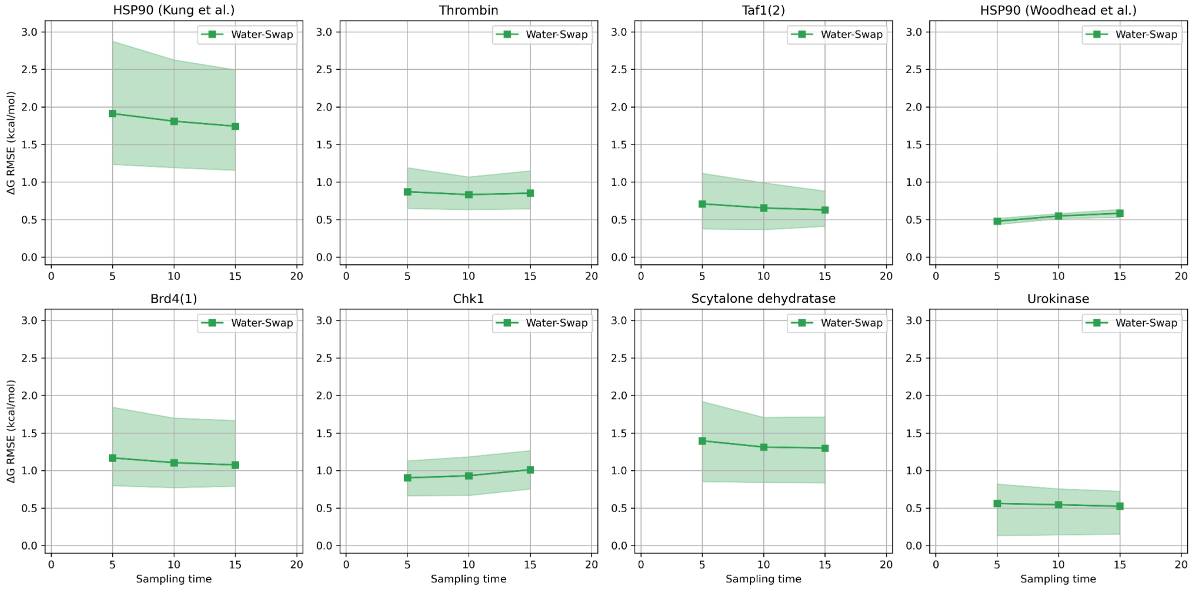

Figure S4.2. RMSE of ΔG for 8 systems simulated with Amber19SB/GAFF2/OPC

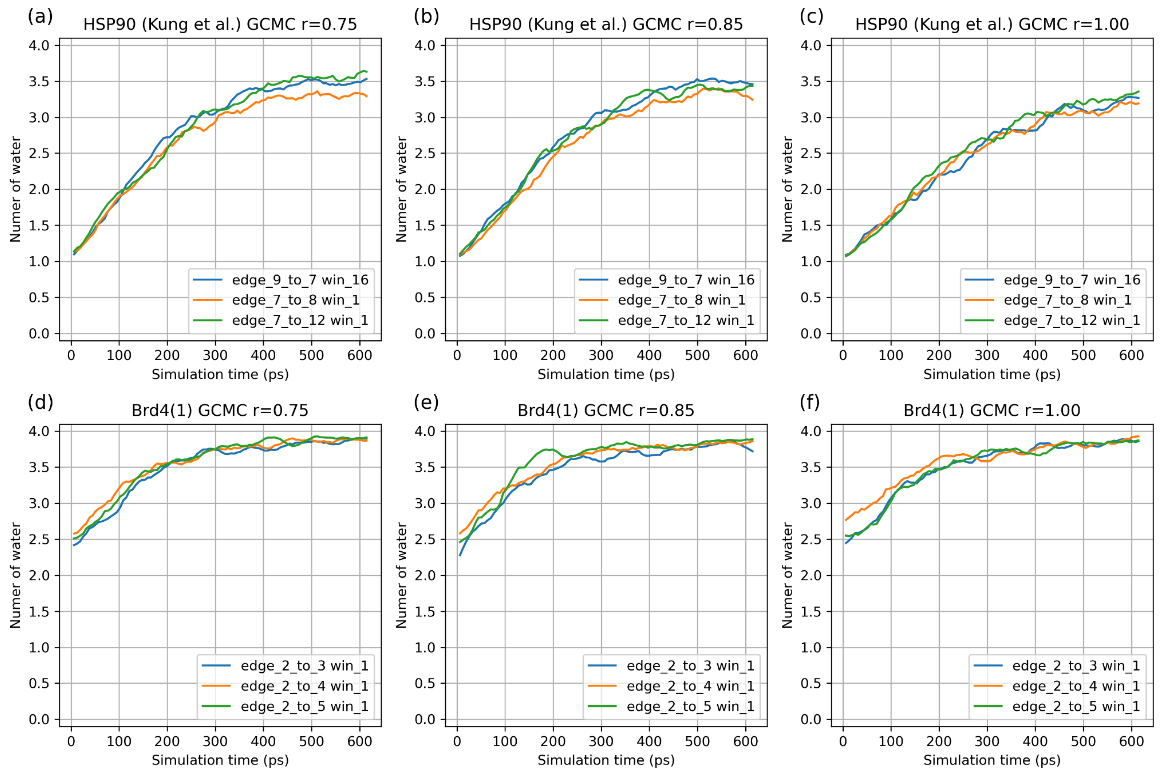

Figure S5.1. Rapid rehydration of conserved binding-site waters by GCMC RBFE (ΔΔG) calculations. Conserved hydration sites in the binding pockets of HSP90 (a, b, c) and Brd4(1) (d, e, f) were rehydrated with GCMC using different active site radius settings. Each time series is averaged over 40 repeats. (b) shows the same data as Figure 2(a), and (e) shows the same data as Figure 2(c).

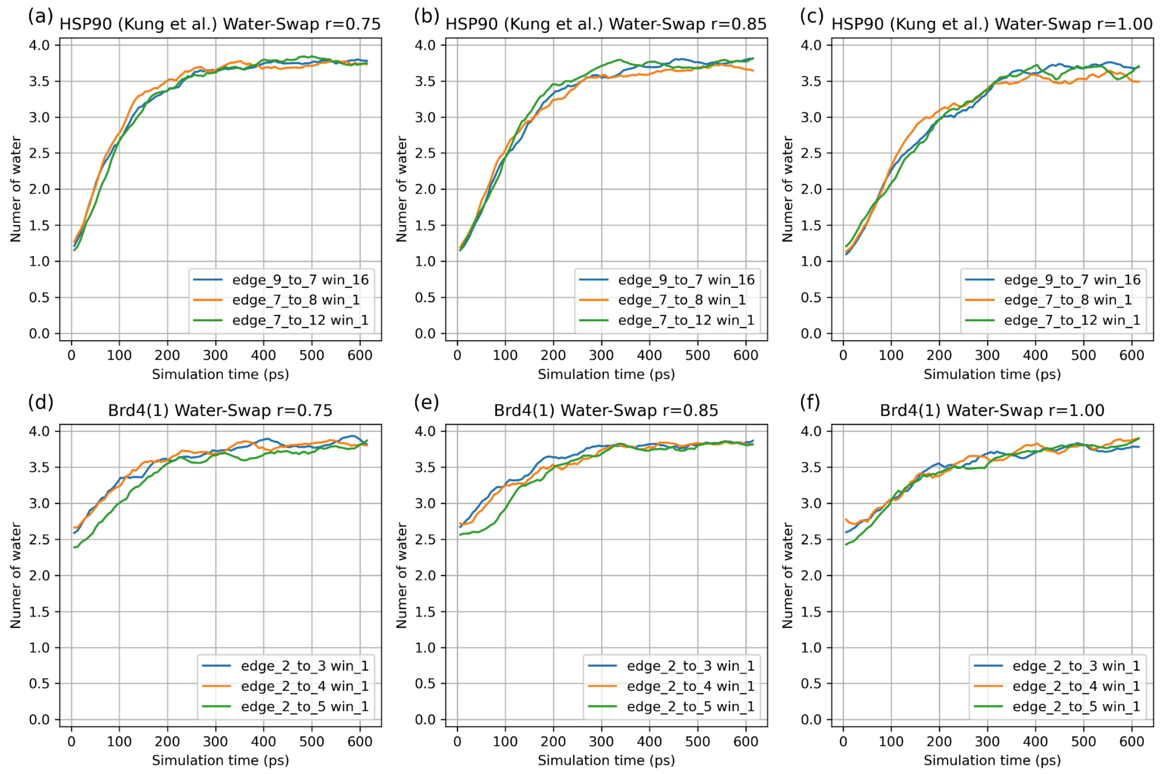

Figure S5.2. Rapid rehydration of conserved binding-site waters by water-swap RBFE (ΔΔG) calculations. Conserved hydration sites in the binding pockets of HSP90 (a, b, c) and Brd4(1) (d, e, f) were rehydrated with water-swap using different active site radius settings. Each time series is averaged over 40 repeats. (b) shows the same data as Figure 2(b), and (e) shows the same data as Figure 2(d).

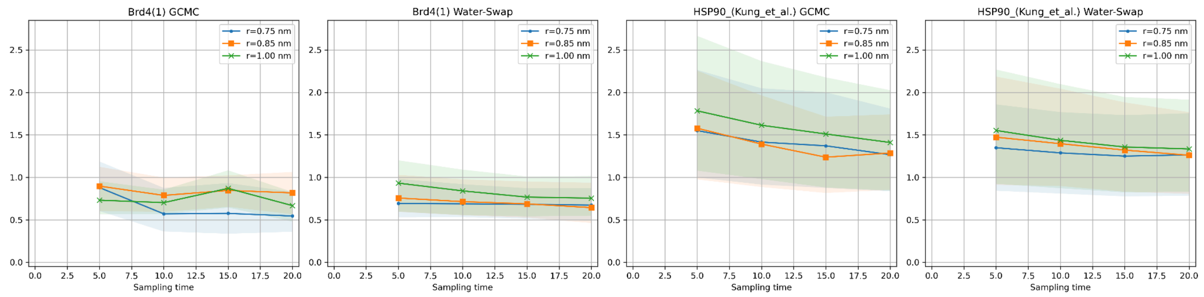

Figure S6. XXX

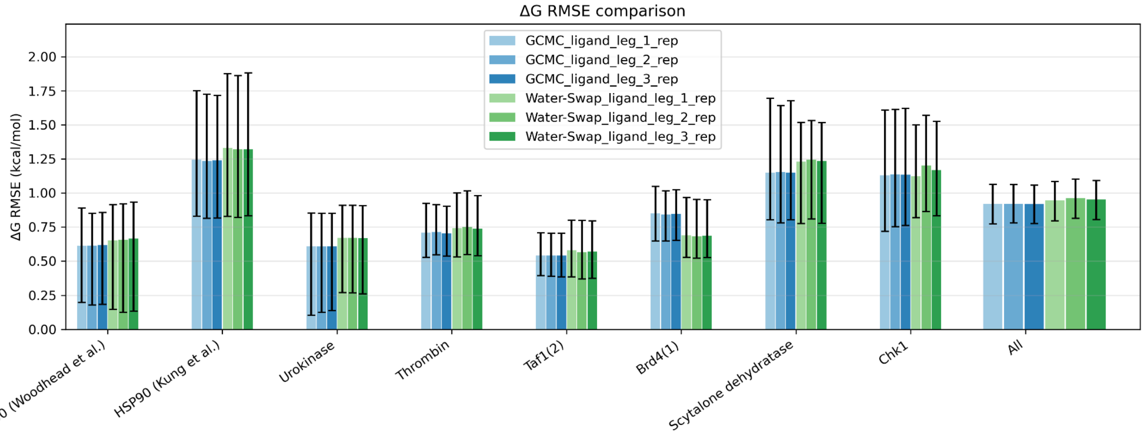

Figure S7. Accuracy between the experimental and predicted binding free energy (ΔG) for 8 systems using GCMC and water-swap MC with ligand leg simulated 1/2/3 repeats.

Table S1. Overall RMSE of binding free energy with ligand leg simulated 1/2/3 repeats

	RMSE (kcal/mol)	95% CI
GCMC_ligand_leg_1_rep	0.92	0.78–1.06
GCMC_ligand_leg_2_rep	0.92	0.78–1.06
GCMC_ligand_leg_3_rep	0.92	0.77–1.06
Water-Swap_ligand_leg_1_rep	0.95	0.80–1.08
Water-Swap_ligand_leg_2_rep	0.95	0.81–1.10
Water-Swap_ligand_leg_3_rep	0.95	0.80–1.09

4. Code Availability

All of the code used in this paper can be found on github. https://github.com/deGrootLab/GrandFEP/
